# Ecological sensitivity and vulnerability of fishing fleet landings to climate change across regions

**DOI:** 10.1038/s41598-022-21284-3

**Published:** 2022-10-17

**Authors:** Marta Albo-Puigserver, Juan Bueno-Pardo, Miguel Pinto, João N. Monteiro, Andreia Ovelheiro, Maria A. Teodósio, Francisco Leitão

**Affiliations:** 1 Centro de Ciências do Mar (CCMAR), Universidade do Algarve, Campus de Gambelas, 8005-139 Faro, Portugal; 2 Centro Oceanográfico de Baleares, Instituto Español de Oceanografía (IEO-CSIC), Ecosystem Oceanography Group, Moll de Ponet sn, 07015 Palma, Spain; 3 Centro de Investigación Mariña, Universidade de Vigo, Future Oceans Lab, Campus Lagoas-Marcosende, 36310 Vigo, Spain

**Keywords:** Climate change, Ocean sciences, Climate-change ecology, Environmental social sciences, Climate-change adaptation

## Abstract

The degree of exposure of fishing communities to environmental changes can be partially determined by the vulnerability of the target species and the landings composition. Hence, identifying the species that ecologically most contribute to the vulnerability of the landings are key steps to evaluate the risk posed by climate change. We analyse the temporal variability in intrinsic sensitivity and the ecological vulnerability of the Portuguese fisheries landings, considering the species proportions derived both from the weights and revenues. To account for the diversification of species of each fleet, we explored the species dependence of the fishery in combination with the vulnerability of them. The analyses were carried out separately for three fleet typologies and three regions. Opposite to what has been observed at a global scale, the ecological sensitivity of the fisheries landings between 1989 and 2015 did not display a decline across areas or fishing fleets. Considering each fleet independently, for trawling, where average vulnerability was lower than in the other fleets, the sensitivity of the landings increased since the 2000s. On the other hand, the high vulnerability found in multi-gear fleets was compensated by diversification of the species caught, while purse-seine fleets targeted low vulnerability species but presented a high fishery dependence on few species. The results highlight the importance of combining information on ecological vulnerability and diversification of fishing resources at a regional scale while providing a measure of the ecological exposure to climate change.

## Introduction

Climate change impacts marine ecosystems at different levels of biological organization, from individuals to populations and ecosystems. Consequently, the goods and services these ecosystems provide to human society are also affected. Among these services, fisheries capture most attention from scientists and managers due to their social and economic relevance^1,2^. Nevertheless, not all fisheries are expected to be equally impacted by climate change. Among other aspects, the exposure to climate change of fishing communities will depend on the ecological vulnerability of the species catch. Hence, from a management point of view, to increase the resilience of fisher communities, it is important to reduce their dependence not only on single resources but also on highly vulnerable species^3–5^.

The vulnerability of species to climate change has been defined as the degree to which the species is susceptible to, or unable to cope with, the adverse effects of climate change. It depends on the level, magnitude and rate of exposure to climate variables (extrinsic factors), its sensitivity to climate change in the sense of the degree to which the population dynamics and life-history traits of a given species are affected by a change in the environment, and its adaptive capacity to respond to climate impacts, whether by adjusting to potential damage or taking advantage of opportunities^6–8^. At a global scale, it has been observed that ecosystems that are highly exploited present a decreasing trend in the landings of species with high sensitivity values^9^. This is due to the fact that fisheries tend to first remove large, slow-growing, long-lived species that, because of their life-history strategies, tend to be more sensitive^10^. To determine the ecological vulnerability of the landings, the sensitivity of species composing the landings should be assessed together with the degree of exposure and the adaptation capacity of the species, which is driven by aspects related to their biology, ecology, conservation status, or management, among others^11–13^.

The diversity of the landed species composition (i.e. species portfolio^14^) has also been described as an important factor to consider when evaluating the resilience and the vulnerability of a fishery. Previous research suggests that fishery managers and fishers should increase diversification of species landed to reduce the dependence of fishers’ activity on single species^15^. Hence, to increase the adaptive capacity of the fisheries to current and future environmental change conditions, the diversification of fisheries should ideally be accompanied by the selection of low vulnerability species in the portfolio^16^. Considering that economic resources for fisheries adaptation management are usually limited, tools for managers to prioritize efforts are needed. Given the large issues that affect marine fisheries socio-ecological system, different proposals for prioritizing management strategies are available in the literature^17,18^, with the reduction of the fishery dependence on vulnerable species being a major consensual issue^5,19^.

In the last decade, different studies have applied climate vulnerability assessments to inform policy-makers at a national level^20–22^. However, more recent studies have underlined the need to consider the spatial heterogeneity of the socio-ecological system at a regional level in the development of climate vulnerability assessments, in countries that present geographical, environmental and socio-ecological gradients^23,24^. This is the case of Portugal, which follows a North–South orientation with strong latitudinal environmental gradients^25–27^, with differentiated oceanographic and geographic characteristics^28^ that influence fisheries differently across the Portuguese coast^29–31^.

Total annual landings in Portugal in 2019 were 184 thousand tonnes and composed 3.3% of the fisheries production of the European Union (EU-27; source Eurostat). Despite the low percentage of production, Portugal is ranked fifth among countries in the EU-27 in terms of people employed in the fisheries industry. The fish consumption per capita is the highest in the EU^32^ and the third highest in the world (about 60.92 kg), being an important source of protein for the Portuguese population^33,34^. At a local scale, fisheries have a high social and cultural importance and are the economic basis of many communities^35^. The resources on the Portuguese coast have been highly exploited for some species^28,36,37^, however not all fisheries are equally exposed to climate change, as their ecological vulnerability depend on the targeted species^38^. Recently, the ecological vulnerability to climate change and its components (exposure, sensitivity and adaptive capacity) of the main fish and invertebrates with economic interest in Portugal have been defined considering three regions of the Portuguese coast: North, Centre and South^39^.

Here, we used the case of the Portuguese fisheries to investigate if long-term changes in landing sensitivity have been observed and if the ecological vulnerability of the landing differs between fishing fleets and areas. Specifically, we first evaluated (i) the long-term trends in the sensitivity of the landings estimates based on life history traits of the species. Assuming that these traits are constant through time, we looked at the temporal variability of the sensitivity of the landings considering annual landings composition proportions per fleet and region (both in terms of weight, Kg, and revenues, €). Considering that at a global scale species with low sensitivity have been shown to dominate the catches because of the overexploitation of the most sensitive species^9^, we hypothesize that in the Portuguese fisheries a similar decline in sensitivity would be observed due to the historically high fishing exploitation. Second, (ii) we studied whether the landings of multi-gear, purse-seine and trawling fleets were equally vulnerable to climate change, also in terms of landing weight composition and economic revenue. Finally, (iii) aiming to establish a basis for defining priority action species, we followed the proposed methodology of Johnson and Welch^7^, which combines the vulnerability of the species with their importance for the fishing community. We modified this methodology to propose a decision-support framework that is based on the combination of the vulnerability of the species and the dependence of the fishers on them, by means of landing weights and revenues of the species. Identifying the species that most contribute to the vulnerability of Portuguese fisheries, in terms of landings weight and economic revenue differentiated by area, together with the degree of dependence of each fishery in specific species, will provide the information needed to determine on which species, fishery sector and region of Portugal management should be prioritized, to reduce the ecological exposure of the fishing community to future climate change effects.

The results from the present study provide, hence, a measure of the ecological exposure of fishery communities to climate change, that can be used in future socio-ecological vulnerability assessments and adaptation plans^5,20^. The use of biological data, with sensitivity and vulnerability indices to climate change in combination with fisheries data is increasingly used to develop climate-informed management frameworks. Accordingly, we argue that the results provided here could be useful for implementing and inform suitable management strategies in the Portuguese fisheries.

## Material and methods

**Figure 1:**
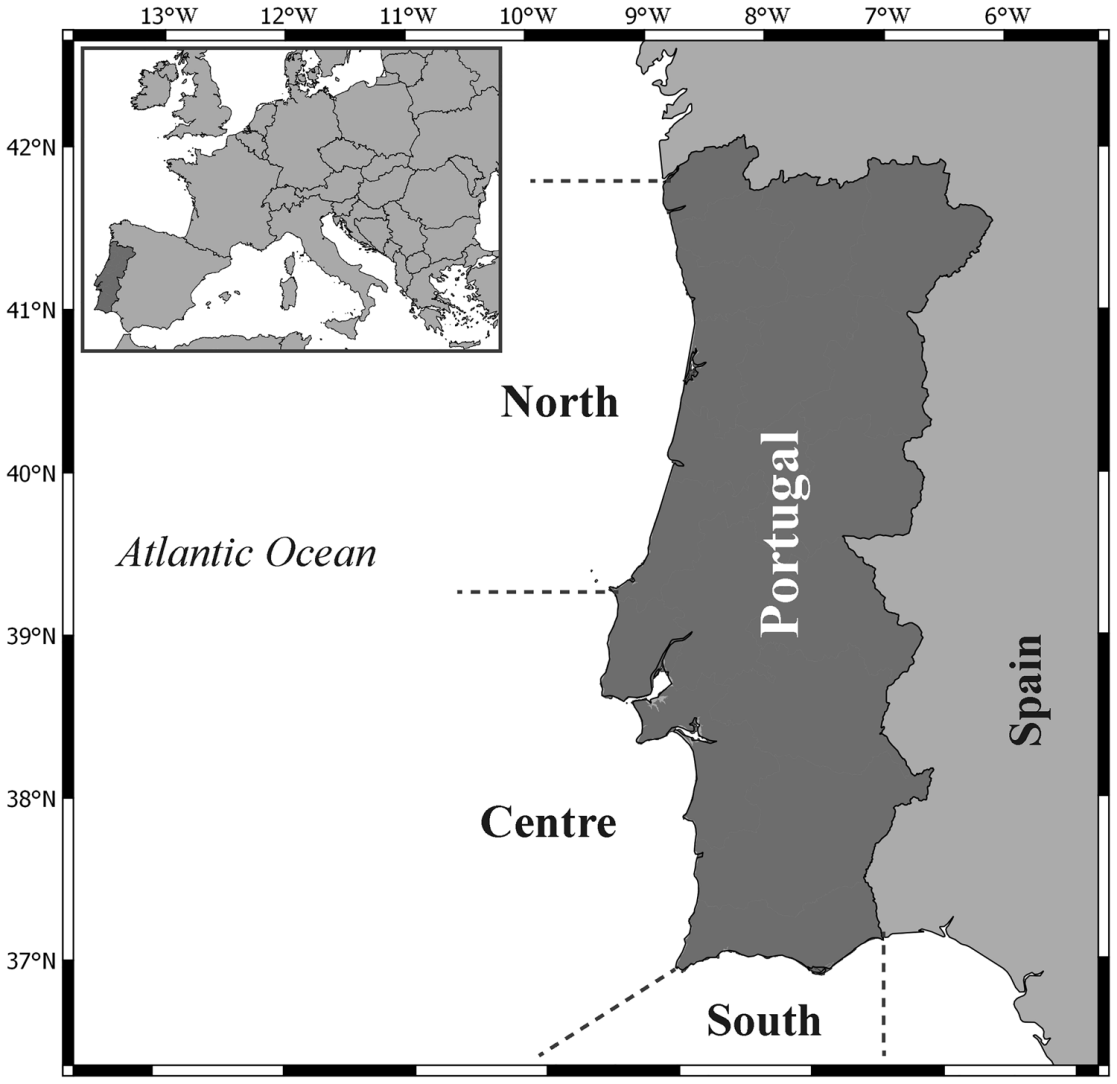
Map of Portugal showing the study area. Horizontal dashed lines delimitate the North, Centre and South area considered in this study. The map was created using QGIS 3.6.3 (http://qgis.org).

Three different fishing fleets (*métiers*) are identified in Portuguese official fishery statistics: i) multi-gear, a fleet mainly composed on artisanal vessels from 5 to 23 m length operating with different licensed gears^40,41^, such as gillnets, trammel nets, longlines and traps, targeting a high variety of species ii) purse-seine, which targets mainly sardine (*Sardina pilchardus*), Atlantic chub and horse mackerel (*Scomber colias* and *Trachurus trachurus*, respectively), and iii) trawling, which targets species such as Atlantic horse mackerel, European hake (*Merluccius merluccius*), cephalopods and crustaceans^28,42^. The study area corresponds to the continental Portuguese coast, which was subdivided into three regions considering the oceanographic and fish assemblage’s characteristics described in previous studies (North, Centre, South^42,43^) (Fig. 1).

Two different data sources were used. Species ecological vulnerability values and its dimensions (exposure, sensitivity and adaptive capacity) of 74 commercial marine species were obtained from Bueno-Pardo et al*.*^39^, under climate change scenario RCP 4.5 and RCP 8.5. These authors estimated the vulnerability of species to climate change was evaluated following the framework of the 4th Assessment Report of the IPCC and the final score of vulnerability for each species was a function of the exposure to the changing environment (RCP 4.5 and RCP 8.5), the sensitivity to the environmental change and the adaptive capacity of the species^6,39^. Each of these components was evaluated considering different indicators that accounted for aspects related to the environmental variables, biology, ecology and exploitation of the species (see Bueno-Pardo et al.^39^, for a full description of the indicators). The second data source was the public data on annual official landing weights(kg) and prices (€/kg) for each species, port and fishing fleet obtained for the period 1989–2015 from the Portuguese National Agency Directorate-General for Natural Resources Safety and Maritime Services (DGRM).

### Trends in the ecological sensitivity of the landings

Species sensitivity to climate change were retrieved from Bueno-Pardo et al*.*^39^. This parameter measures the extent to which the population dynamics or life history traits of a given species will be affected by changes in the environment (i.e. fecundity, growth parameters, age at maturity, planktonic larval duration, among others). Similarly, in previous works, this sensitivity parameter was called “intrinsic vulnerability” and since changes in life history traits occur in the long term, sensitivity is considered to be constant through time and is independent of the climate change scenario^9^. The sensitivity values reported for the main commercial species in combination with annual official landing data were used to explore temporal changes in ecological sensitivity of the landings from 1989 to 2015.

The Ecological Sensitivity of the Landing composition (ESL), in terms of biomass, was calculated and inferred from the average sensitivity of each of the exploited species, weighted by their annual landings, as follows:

1 $${\text{ESL}} = \mathop \sum \limits_{i}^{n} \frac{Li,yr* Si}{{TLyr}}$$

where *L*_i_ is the landing weight of the species *i* for the year (*yr*), *S*_i_ is the sensitivity value estimated by Bueno-Pardo et al*.*^39^ for the species *i*, TL_*yr*_ is the total landings of that year considering all the species and *n* is the total annual number of landed species. High values of ESL represented a high proportion of very sensitive species in the landings. The yearly ESL was calculated for each area (north, centre and south) and fleet sector (multi-gear, purse-seine, trawling).

To capture general patterns in the temporal trends of changes in sensitivity of the landings for each fishing fleet and area, the ESL was plotted using the “ggplot” package^44^ with a locally weighted regression smoother (*loess* smoother), to reduce the variability and better reflect the underlying long-term trend. The *loess* smoother was fitted using the default span (0.75) of the R software^45^. Additionally, a Dynamic Factor Analysis (DFA) was applied to test if there was a common declining trend in the ESL, within fishing fleets and areas. DFA is a multivariate time series analysis technique used for non-stationary time series analysis which allows us to estimate underlying common trends. A diagonal and unequal error covariance matrix was used for the models, and the corrected Akaike Information Criterion (AICc) was used to compare the models^46^. We used the model given by N time series = linear combination of M common trends + noise. Canonical correlations among time series were used to indicate either a positive or negative relationship. Correlations with an absolute value higher than 0.5 indicate a significant relationship between variables^47^. Response variables were normalized (mean subtracted, divided by standard deviation) prior to the analysis. Data analysis was performed using the software package Brodgar 2.7.5 (www.brodgar.com), considering 5000 iterations for each model. To determine which species contributed the most to the changes in ESL, the sensitivity, weight and landings of the main species contributing more than 3% were plotted (see Supplementary Fig. S1–S3 online).

### Ecological vulnerability of the landings

The assessment of the ecological vulnerability of the landings of each fishing fleet and area of Portugal was performed considering the vulnerability of commercial species determined in Bueno-Pardo et al., (2021) for the two scenarios of climate changes (RCP 4.5 and RCP 8.5). The vulnerability values were used in combination with the landing composition of the fishing fleet in terms of landing weight (kg) and economic revenue of the landings (€). Available landing data for the most recent period (2010–2015) was selected as a proxy of the average landing composition, to calculate the actual mean ecological vulnerability of the landings. The ecological vulnerability of the landings was calculated for each year from 2010 to 2015 and then the average of all the years was used as ther mean overall ecological vulnerability of the landings.

The Ecological Vulnerability of the Landings, considering weight (EVL_kg_), was calculated and inferred from the cumulative contribution of the vulnerability of each of the exploited species for each scenario, weighted by their landings, for each year for the period 2010–2015, as follows:

2 $$\mathop \sum \limits_{2010}^{2015} {\text{EVL}} = \mathop \sum \limits_{i}^{n} \frac{Li, yr *Vi}{{TL yr}}$$

In Eq. (2), *L*_i_ is the landing weight of the species *i* for the year (*yr*), TL_*yr*_ represents the total landings for the year considering all the species, V_i_ is the vulnerability estimated by Bueno-Pardo et al*.*^39^ for the species *i* and *n* is the total number of landed species. The EVL_(kg)_ was calculated yearly for the period 2010–2015 and averaged to obtain the final EVL_(kg)_ value. Higher values of EVL_(kg)_ represented a higher proportion of species with high vulnerability in the landings. The EVL_(kg)_ was estimated separately for each fleet sector (multi-gear, purse-seine and trawling), area (north, centre and south) and climate change scenario (RCP 4.5 and RCP 8.5).

Similarly, to obtain the EVL based on economic revenue, the mean price of each species in nominal value (€/kg) was obtained yearly from the DGRM, and was multiplied by the landings of the species, to obtain the total economic revenue by species. Then, instead of using L*i* (in kg) in Eq. (2), the revenue of each species and the total revenue (R*i*) of the area and gear type was used to calculate the EVL_(€)_ based on landing revenue. The value of inflation between years was not considered, due to the short period included in the analysis (2010–2015). Revenues are expressed in nominal values (euros).

Since vulnerability of species is the sum of both sensitivity and exposure minus the adaptive capacity^39^, we explored the analysis of each component of the vulnerability separately. The same procedure was used to calculate the mean exposure, sensitivity and adaptive capacity of the landings for the period 2010–2015, substituting the vulnerability values of each species (V*i* in Eq. 2) with the corresponding values of exposure (E*i*), sensitivity (S*i*) and adaptive capacity (AC*i*) by species and area reported in Bueno-Pardo et al*.*^39^. Since values of exposure of each species were reported for both scenarios^39^, the ecological exposure of the landings was calculated for the RCP 4.5 and RCP 8.5. The average and standard deviation of the period 2010–2015 was calculated for each component and scenario.

Differences in EVL between areas, fleets and scenarios were tested by using a two-way analysis of variance (ANOVA test) followed by Tukey’s hsd post-hoc test (aov, base package^45^). The assumptions of ANOVA were checked with a Kolmogorov–Smirnov test for normality and a Levene’s test for homogeneity of variances. In the case ANOVA assumptions were not met the non-parametric test Kruskal–Wallis was used to compare the mean ecological exposure, sensitivity and adaptive capacity of the landings between fishing fleets and areas. The threshold for significance was α < 0.05. The proportion contributed by each species to the total EVL was calculated in both landings weight and revenue. Only species contributing more than 3% to the EVL were reported. Additionally, an analysis of similarity percentages (SIMPER) was applied to determine the average dissimilarity in species composition between areas for each fleet sector separately and to identify the main species contributing to the dissimilarity. Bray–Curtis dissimilarity was applied, and SIMPER analysis was done using PRIMER-E 7 software^48^.

### Prioritization of species for management

An analysis to identify which species present higher vulnerability and represent an important fraction of landed fish in weight and economic revenue was conducted adapting the methodology proposed in Johnson and Welch^7^. Species’ vulnerability score was classified and obtained from Bueno-Pardo et al*.*^39^. The following categories of vulnerability were used: very low (< 0.20), low (0.20–0.40), moderate (0.40–0.60), high (0.60–0.80), and very high (> 0.80). The degree of “fisheries dependence” on each target species was defined considering the contribution (%) of each species to the total landings in terms of weight (kg) and revenue (€). Both parameters were averaged to obtain a single index of fisheries dependence for each landed species and was classified in the following categories: very low (< 5% contribution), low (5–15%), moderate (15–30%), high (30–50%), and very high (> 50%). Only species contributing more than 3% to the total vulnerability and/or to the total landings weight and landing revenue were considered. To provide a visual frame of decision-support for selecting which species to target for priority action, a scatter plot (prioritization plot) with the classification of the vulnerability, and fisheries dependence on species was represented. High levels of dependency are indicative of a low degree of species diversification of the fishing fleet, while low levels of dependence indicate a higher degree of species diversification. The categorization of vulnerability and fisheries dependence aim to facilitate the visualization of the results, but should be interpreted as relative values comparing results of this study.

## Results

### Trends in sensitivity of the landings

Landings of purse-seine had lower values of sensitivity than multi-gear and trawling between 1989 and 2015 for the three areas. Sensitivity values of purse-seine landings were between 0.321 and 0.342, whereas multi-gear and trawling landings in most years had values above 0.4, with sensitivities between 0.364 and 0.513, and 0.355 and 0.489 for multi-gear and trawling, respectively.

**Figure 2:**
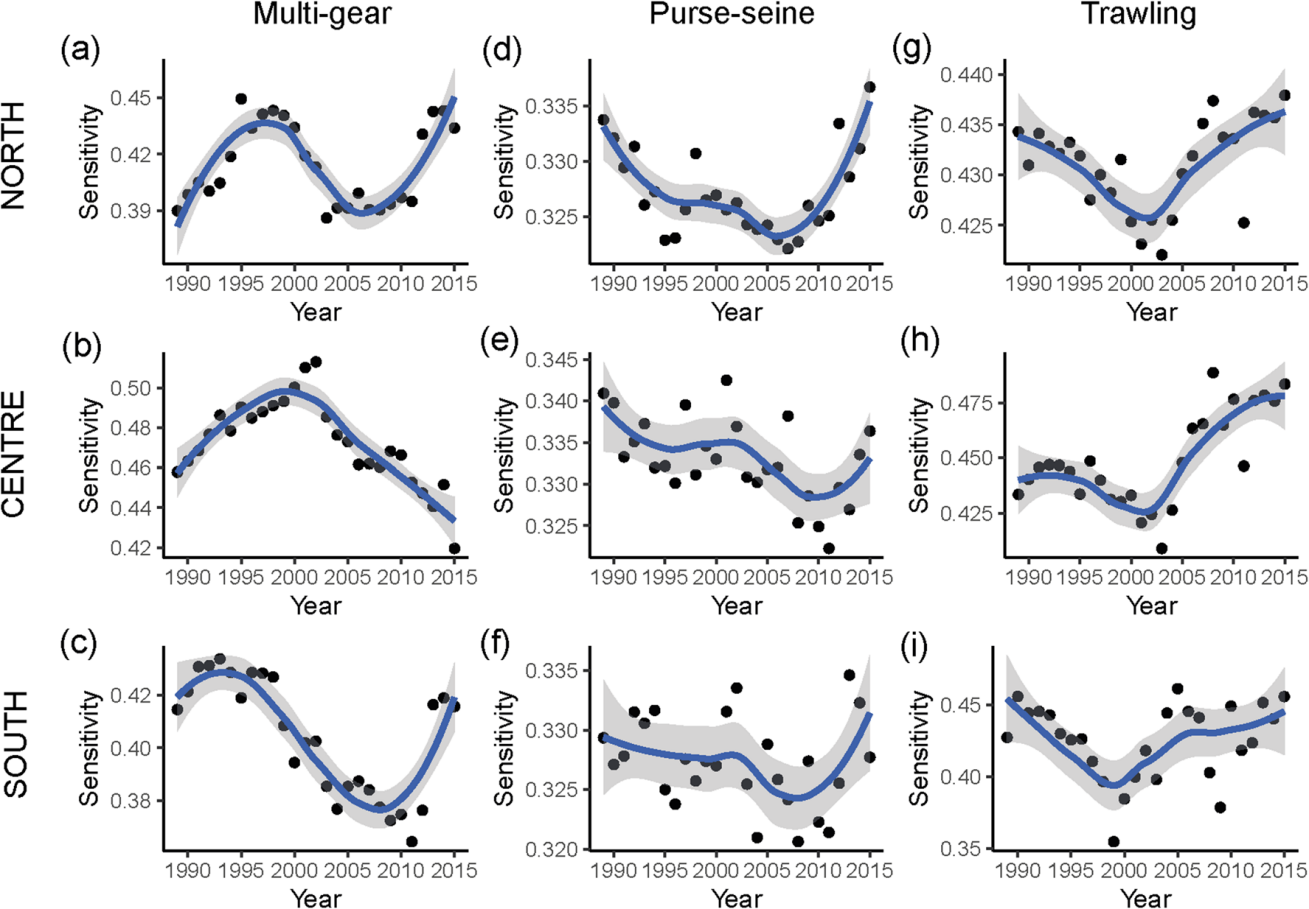
Ecological Sensitivity of the Landing (ESL) time series (dots) and temporal trends from 1989 to 2015 of each fishing fleet and area; Multi-gear (**a**-North; **b**-Centre; **c**-South), purse-seiners (**d**-North; **e**-Centre; **f**-South), and trawlers (**g**-North; **h**-Centre; **i**-South). A local polynomial regression (LOESS) fit is shown in blue, with 95% confidence intervals as a grey shadow.

**Figure 3:**
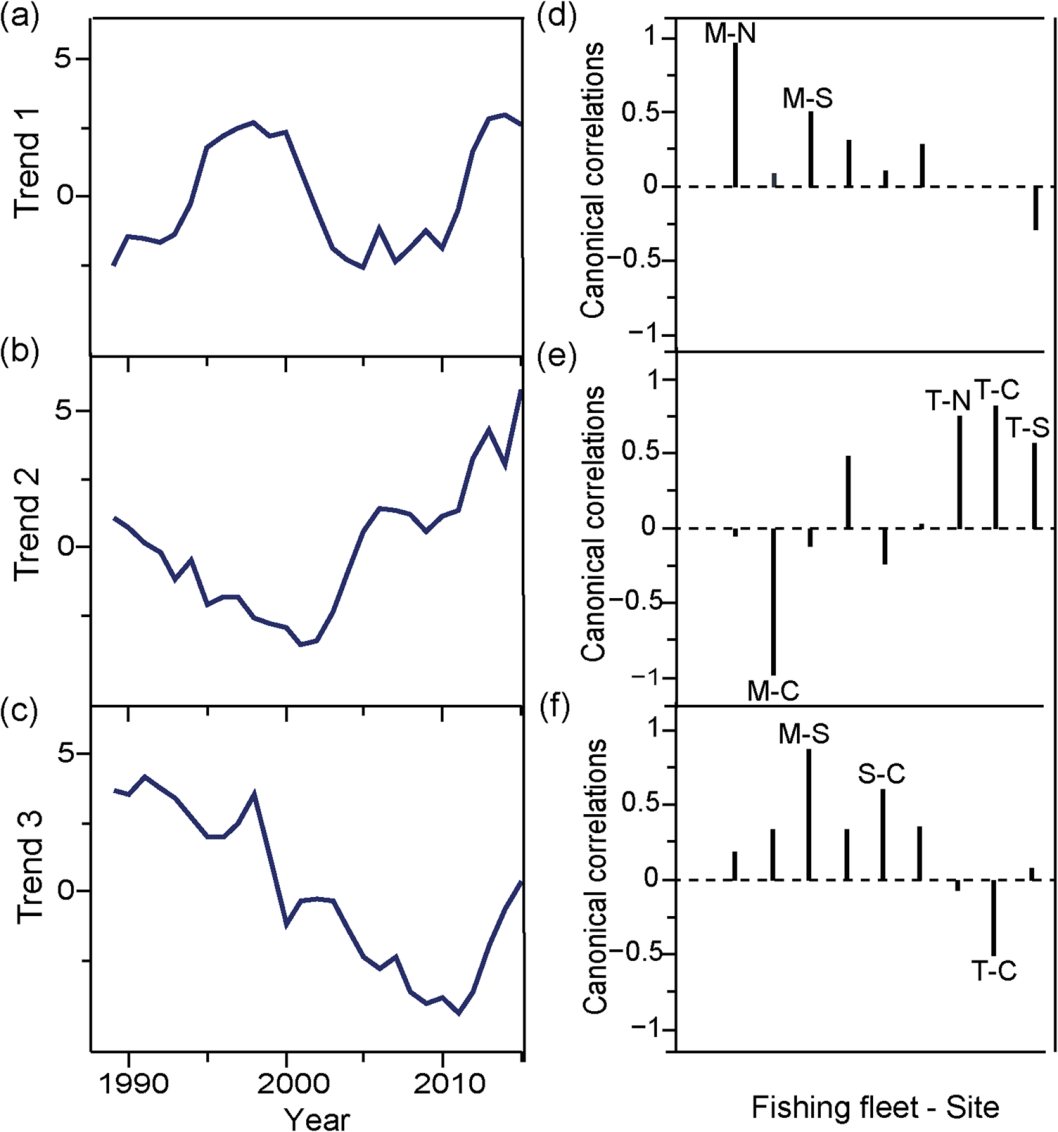
Common trends (left; **a**, **b**, **c**) and the corresponding canonical correlations (right; **d**, **e**, **f**) for the ecological sensitivity of the landings (ESL) series obtained by the model containing three common trends. Common trends are unitless. Labelled correlations correspond to the significant correlations (> 0.5). The first letter of the abbreviation corresponds to the fishing fleet (M = Multi-gear; S = purse-Seine; T = Trawling) and the second letter to the area (N = North; C = Centre; S = South).

We observed different temporal patterns between areas within each fleet (Fig. 2 and 3). The sensitivity for multi-gear in the north shift across years and the periods with higher sensitivity values corresponded mainly to the decrease in landings of *Sardina pilchardus* (Fig. 2a; Supplementary Fig. S1 online). In the centre area, maximum values of sensitivity in the period 2000–2002 corresponded to the decline in landings of *Lepidopus caudatus* from 1997, and to the increase in species with higher sensitivity, such as *Aphanopus carbo* and *Centroscymnus coelolepis* (Fig. 2b; Supplementary Fig. S1 online). From 2003, the decline of ESL was associated with the increase in landings of species with lower sensitivity, such as *Scomber colias*. In the south area, the decline in ESL in the period 1995–2012 was explained by the combination of a decline in contribution of species with a sensitivity score higher than 0.45, such as *Merluccius merluccius*, *Lepidopus caudatus* and *Conger conger* and the increase in species with a lower sensitivity such as *S. colias* (Fig. 2c; Supplementary Fig. S1 online). The only increase in ESL was recorded between 2013 and 2015 due to the increase in landings of *Octopus vulgaris* and the decline of *S. pilchardus* and *S. colias*.

For purse-seiners the ESL was similar in the three areas and presented low values (sensitivity values lower than 0.35) for the entire period (Fig. 2d,f). The main species that contributed to the ESL were *S. pilchardus*, followed by *S. colias* (Fig. 2; Supplementary Fig. S1 online). In the north area, the dominance of *S. pilchardus* was maintained until 2011 when the proportion of sensitivity explained by *S. colias* and *Trachurus trachurus* increased due to the decline in landings of *S. pilchardus* and the increase in *S. colias* (Fig. 2d; Supplementary Fig. S2 online). In the centre and south areas, a decline in the contribution of *S. pilchardus* to the ESL started in 2005, coinciding with the increase in *S. colias* landings (Fig. 2e,f; Supplementary Fig. S2 online).

Trawling ESL increased in the three areas from 2000 or 2003 onwards (Fig. 2). The main species contributing to the sensitivity in the north was *T. trachurus* (Fig. 2g, Supplementary Fig. S3 online), while in the centre and south areas, ESL variability was mainly affected by the decline of *T. trachurus* and the increase of *Micromesistius potassou* from 2003 onwards (Fig. 2h,i), and the increase of *Parapenaeus longirostris*, between 1997 and 2003 in the south area (Fig. 2i, Supplementary Fig. S3 online).

No common trends in ESL were observed across areas or fishing fleets. Results from DFA revealed three different trends (Fig. 3; Supplementary Table S1 online). Trend 1 is oscillatory and better represents multi-gear ESL time series scores in the north and south area (Fig. 3a,d; Supplementary Table S1 online). Trend 2 reveals a decrease in sensitivity until 2001 increasing onwards. This trend was positively correlated with the trawling fleet in the three areas and presented a negative correlation with multi-gear fleets in the centre area (Fig. 3b,e). Trend 3 presented a clear decline in ESL until 2011 and the higher positive correlations with this trend were observed in multi-gear fleets in the south and purse-seine fleets in the centre area (Fig. 3c,f; Supplementary Table S1 online).

### Ecological vulnerability of the landings

**Figure 4:**
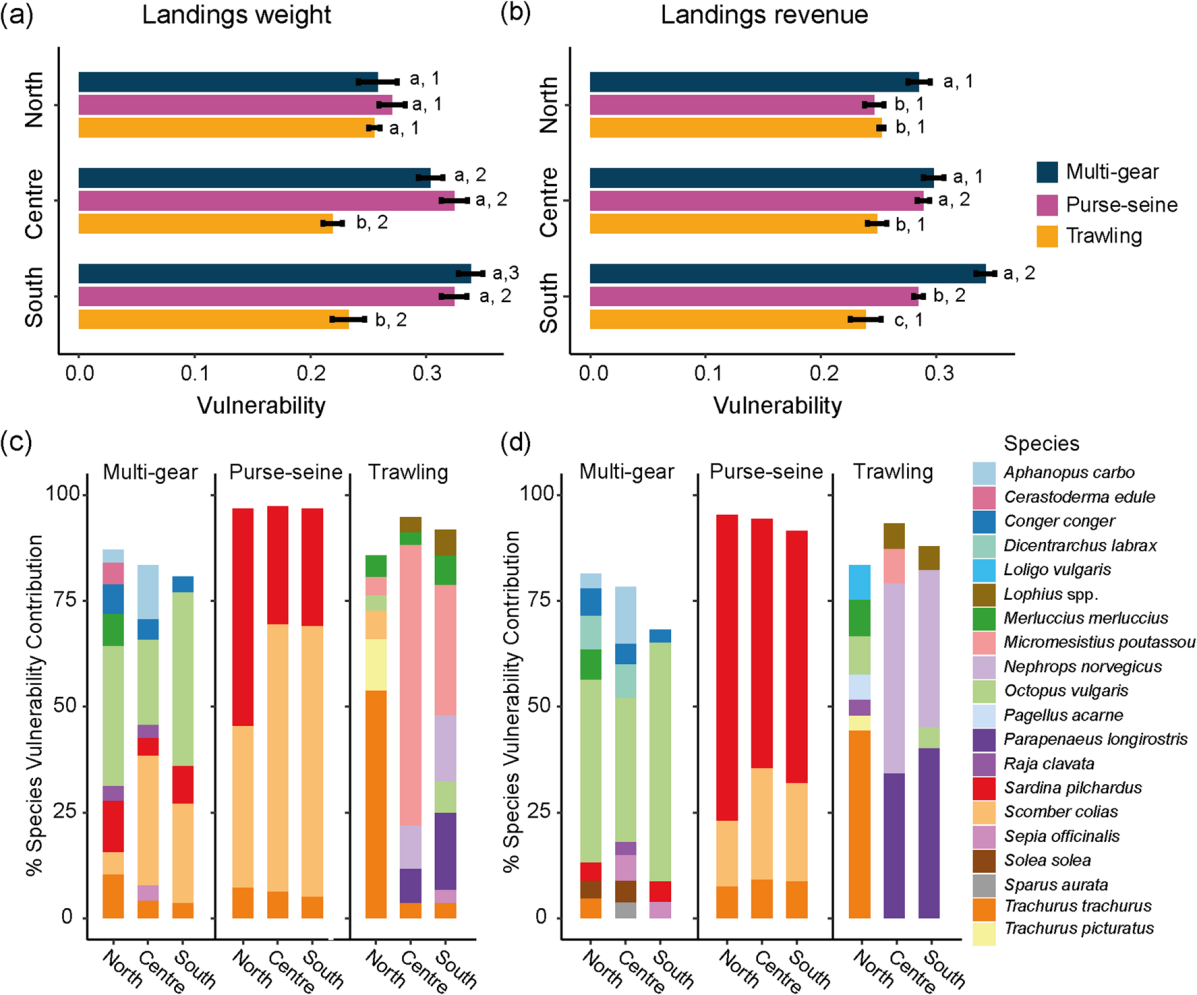
Ecological vulnerability of the landings weight (**a**) and economic revenue (**b**) by gear type and area calculated for the average period 2010–2015 and RCP 8.5. Each gear type is represented with a different colour. Tukey’s HSD Test for multiple comparisons between fleets within the same area are indicated with letters and comparisons between areas within each fleet sector are indicated with a number. Pairs that were similar are indicated with the same number or letter and pairs that were significantly different (p < 0.05) are indicated with a different numer or letter. The contribution of each species to the total ecological vulnerability of the fleet in terms of landings weight (**c**) and landings revenue (**d**) is represented for each gear type (Multi-gear; Purse-seine; Trawling). Only species contribution with > 3% to the total vulnerability are represented in the graph.

The ecological vulnerability of the landings was significantly different between areas (kg; *F*_(2,45)_ = 47.47, p < 0.001) and fleets (kg; *F*_(2,45)_ = 208.09, p < 0.001) in terms of weight and economic revenue (€; *F*_area (2,45)_ = 50.27, p < 0.001; *F*_fleet (2,45)_ = 255.35, p < 0.001). Multi-gear and purse-seine fleets in the centre and south areas presented the highest EVL in weight (Fig. 4). The highest ecological vulnerability of the landings, according to the revenues, was found for multi-gear fleets in the south. Values of ecological vulnerability and exposure were slightly higher in the climate change scenarios RCP 8.5 (Table 1). Thus, considering the similarity between both scenarios, results based on the RCP 4.5 are reported in the supplementary material.

**Table 1:** Mean and standard deviation values of ecological vulnerability (V), exposure (E), sensitivity (S) and adaptive capacity (AC) of the landings weight and landings revenues for each area (North, Centre and South), fleet (multi-gear, purse-seine and trawling) and climate change scenario (RCP 4.5 and RCP 8.5).

Fleet		Multi-gear		Purse-seine		Trawling	
Scenario		RCP 4.5	RCP 8.5	RCP 4.5	RCP 8.5	RCP 4.5	RCP 8.5
**Landings weight**							
North	V	0.250 ± 0.017	0.258 ± 0.017	0.262 ± 0.011	0.270 ± 0.011	0.247 ± 0.005	0.255 ± 0.005
	E	0.472 ± 0.013	0.480 ± 0.013	0.508 ± 0.001	0.517 ± 0.001	0.459 ± 0.005	0.466 ± 0.005
	S	0.424 ± 0.022		0.330 ± 0.005		0.434 ± 0.016	
	AC	0.645 ± 0.028		0.577 ± 0.010		0.645 ± 0.011	
Centre	V	0.278 ± 0.010	0.304 ± 0.011	0.297 ± 0.011	0.324 ± 0.011	0.195 ± 0.008	0.219 ± 0.008
	E	0.453 ± 0.012	0.479 ± 0.012	0.517 ± 0.002	0.545 ± 0.002	0.404 ± 0.010	0.428 ± 0.010
	S	0.446 ± 0.016		0.329 ± 0.05		0.473 ± 0.013	
	AC	0.622 ± 0.013		0.549 ± 0.010		0.682 ± 0.012	
South	V	0.322 ± 0.010	0.338 ± 0.011	0.308 ± 0.011	0.324 ± 0.011	0.219 ± 0.014	0.233 ± 0.014
	E	0.511 ± 0.005	0.527 ± 0.005	0.529 ± 0.002	0.545 ± 0.002	0.428 ± 0.007	0.441 ± 0.007
	S	0.394 ± 0.025		0.327 ± 0.05		0.440 ± 0.016	
	AC	0.582 ± 0.016		0.547 ± 0.011		0.648 ± 0.008	
**Landings revenue**							
North	V	0.277 ± 0.010	0.285 ± 0.010	0.238 ± 0.008	0.246 ± 0.008	0.245 ± 0.002	0.253 ± 0.002
	E	0.463 ± 0.006^a^	0.471 ± 0.006	0.510 ± 0.002	0.519 ± 0.002	0.450 ± 0.002	0.458 ± 0.002
	S	0.477 ± 0.010		0.330 ± 0.002		0.435 ± 0.004	
	AC	0.633 ± 0.013		0.602 ± 0.011		0.640 ± 0.005	
Centre	V	0.271 ± 0.008	0.298 ± 0.009	0.262 ± 0.005	0.289 ± 0.005	0.224 ± 0.008	0.249 ± 0.008
	E	0.451 ± 0.007	0.477 ± 0.008	0.514 ± 0.003	0.541 ± 0.003	0.437 ± 0.005	0.462 ± 0.005
	S	0.478 ± 0.006		0.337 ± 0.004		0.409 ± 0.009	
	AC	0.658 ± 0.007		0.589 ± 0.006		0.623 ± 0.005	
South	V	0.326 ± 0.008	0.343 ± 0.008	0.269 ± 0.004	0.285 ± 0.004	0.225 ± 0.013	0.239 ± 0.013
	E	0.505 ± 0.003	0.522 ± 0.003	0.526 ± 0.003	0.542 ± 0.003	0.449 ± 0.003	0.464 ± 0.003
	S	0.424 ± 0.007		0.339 ± 0.006		0.388 ± 0.013	
	AC	0.603 ± 0.004		0.597 ± 0.005		0.612 ± 0.003	

### EVL–landings weight

The EVL_(kg)_ of multi-gear and purse-seine fleets were higher in the south and centre areas, whereas for trawling the vulnerability was higher in the north area (Fig. 4a, Table 1). Tukey’s multiple comparison HSD Test revealed that the three fleets had similar EVL_(kg)_ in the north, whereas in the centre and south area trawling had significantly lower EVL_(kg)_ than the other two fleet sectors (Fig. 4a, Supplementary Fig. S4 online).

For each fleet, differences observed between areas were explained by dissimilarities in the species composition that most contributed to the EVL_(Kg)_. SIMPER analysis for multi-gear fleets showed higher dissimilarities in species contributing to the vulnerability between the north and the other two areas (Supplementary Table S2 online). *S. colias*, contributed the most to the dissimilarity between the north and the centre and south areas. Also, *Cerastoderma edule* contributed to the lower EVL_(kg)_ values in the north. Dissimilarities between the south and centre areas were mainly explained by the higher contribution of *A. carbo* in the centre area and the higher proportion of *O. vulgaris* in the south area (Fig. 4a,c).

The EVL_(kg)_ of purse-seine fleets was associated with a small number of species and dissimilarities between areas was low (Fig. 4a; Supplementary Table S3 online). SIMPER analysis for purse-seine showed lowest dissimilarity values between the centre and south areas. In the north, the main species contributing to purse-seine vulnerability was *S. pilchardus*, whereas in the centre and south areas the main species was *S. colias* (Fig. 4c). These differences in species contribution to EVL_(kg)_ of purse-seine fleets explained the higher dissimilarity found between north area compared to both centre and south areas (see Supplementary Table S3 online). For trawling, high dissimilarities in the species contributing to vulnerability were observed between north and centre, and north and south areas (Supplementary Table S4 online). The main species that contributed to the dissimilarity between areas for EVL_(Kg)_ were *T. trachurus* in the north, *M. poutassou* in the centre and south areas, followed by *P. longirostris* and *N. norvegicus* (Fig. 4c). In the south, *O. vulgaris* was the species most contributed to the dissimilarity with the centre area (Supplementary Table S4 online).

**Figure 5:**
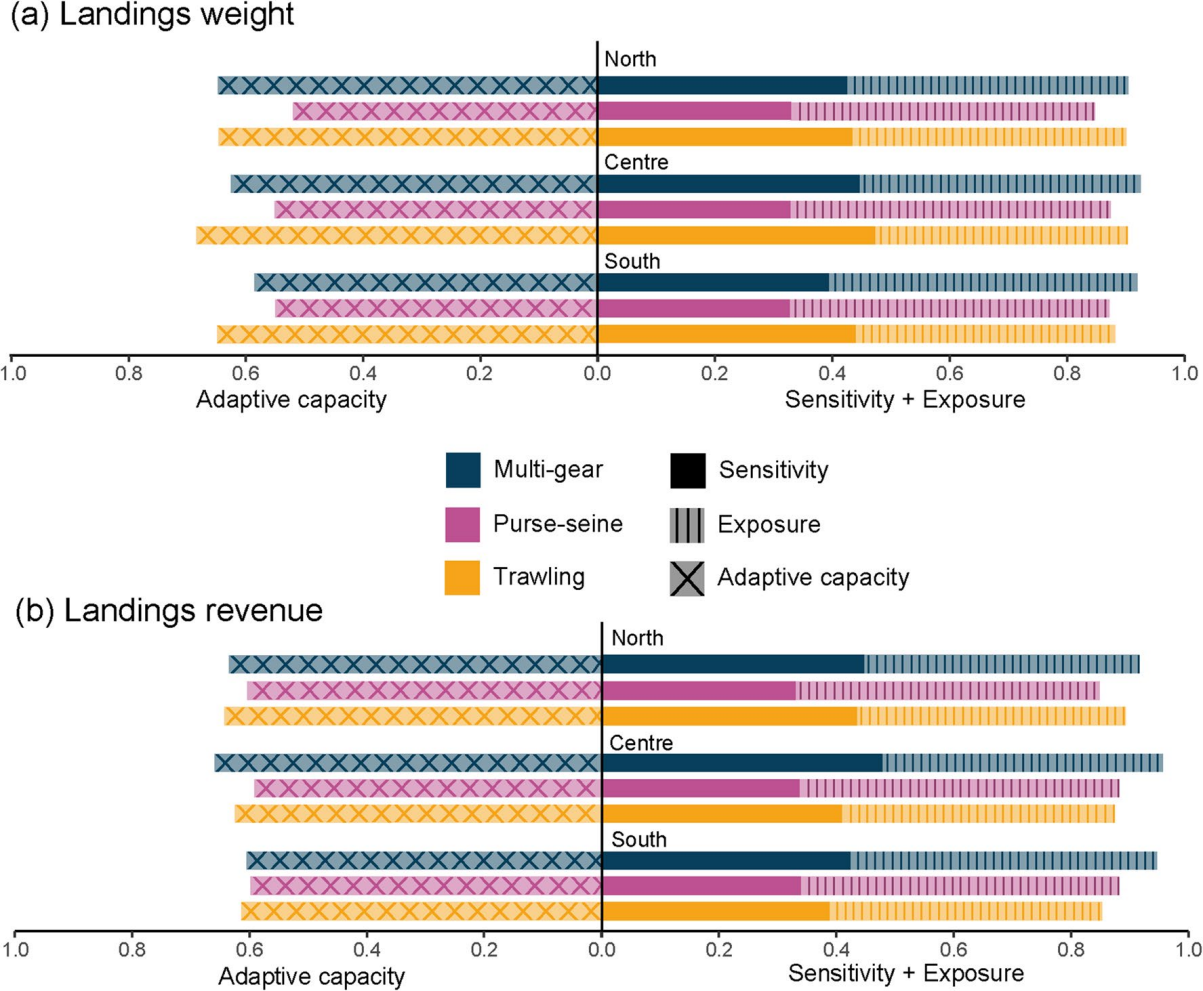
Values of the vulnerability components for the period 2010–2015 (sensitivity + exposure − adaptive capacity). Adpative capacity is represented in the left of the *y* axis, while sensitivity and exposure are represented in the right side of the *y* axis. Each component is represented with a different texture. All components are represented by gear type (colour) and area (north, centre and south). For exposure values correspond to the RCP 8.5. In the top panel the components in terms of landings weight are represented (**a**) and in the bottom panel the components in terms landings economic reveue are represented (**b**).

When analysing the components of the EVL_(kg)_ (sensitivity, exposure and adaptive capacity), we found that the fleet with the highest sensitivity was trawling (Supplementary Table S5 online), whereas purse-seine had the lowest sensitivity values, in the three areas (Fig. 5a; Supplementary Table S6 online). For exposure, an inverse pattern was observed when compared to sensitivity. Purse-seine landings had the highest values of exposure in all areas, followed by multi-gear and trawling (Fig. 5a). The ecological adaptive capacity was higher in trawling landings in the centre and south, whereas in the north multi-gear and trawling landings presented similar adaptive capacity. In the three areas, purse-seine landings had the lowest adaptive capacity (Fig. 5a).

### EVL–landings revenue

The ecological vulnerability of fisheries in revenue (EVL_(€)_) differed from the EVL_(kg)_. We observed an increase in the importance of high price species, such as *O. vulgaris* in multi-gear, *S. pilchardus* in purse-seine and *P.longirostris* or *Nephrops norvegicus* in trawling (Fig. 4d). The EVL_(€)_ of multi-gear was higher in the south, while the purse-seine showed similar EVL_(€)_ in the central and south areas. Trawling did not present differences between areas (Fig. 4b). In the north and south, multi-gear had the highest EVL_(€)_, while trawling had a significantly lower EVL_(€)_ in the centre and south areas (Fig. 4b, Supplementary Fig. S4 online).

For multi-gear, an increase in the contribution of *O. vulgaris* in terms of economic revenue was observed in comparison to the contribution of landing weights (Fig. 4c,d). This is due to the higher average price of *O. vulgaris* (4.33 €·Kg^−1^) compared to *S. pilchardus* (1.57 €·Kg^−1^) and *T. trachurus* (1.60 €·Kg^−1^). Dissimilarity in EVL_(€)_ was higher between the north and south area (Supplementary Table S3 online). *A. carbo* contributed the most to the dissimilarity between areas followed by *O. vulgaris*, which contributed to the dissimilarity of the south area (Fig. 4d).

The species that contributed the most to the EVL_(€)_ in the purse-seine fleet was *S. pilchardus*. The proportion of contribution of *S. pilchardus* in EVL_(€)_ increased in comparison to the contribution to landing weights, due to the higher average price of *S. pilchardus* (1.57 €·Kg^−1^) compared to *S. colias* (0.29 €·Kg^−1^). Dissimilarities in EVL_(€)_ of purse-seine fleets were low between areas, and *S. colias* contributed the most (Supplementary Table S4 online). Similar to multi-gear and purse-seine fleets, the trawling fleets EVL_(€)_ presented differences across areas. While in the north, *T. trachurus* was the main species, in the centre and south area, *P. longirostris* and *N. norvegicus* contributed the most to the EVL_(€),_ due to their high average price (15.66 €·Kg^−1^ and 15.33 €·Kg^−1^, respectively) (Fig. 4D). *M. poutassou* showed a low contribution in terms of economic revenue due to its low average price (0.57 € Kg^−1^)*.* High dissimilarity in EVL_(€)_ was observed between the north and the two other areas, whereas the dissimilarity between the centre and south areas was low (Supplementary Table S6 online).

The sensitivity, exposure and adaptive components of the EVL_(€)_ (Fig. 5b) showed patterns different from those calculated in terms of EVL_(Kg)_. The sensitivity of multi-gear landings revenue was higher in the centre and south area, whereas in the north, multi-gear and trawling presented similar sensitivity values. Purse-seine landings revenue presented the lower sensitivity values in the three areas. For exposure, purse-seiner landings revenue had the highest values among the three areas. The ecological adaptive capacity of multi-gear and trawling landings revenues were higher than in purse-seine fleets in the three areas, although differences in the south were minimal (Fig. 5b; Supplementary Table S6 online).

### Prioritization of species for management

**Figure 6:**
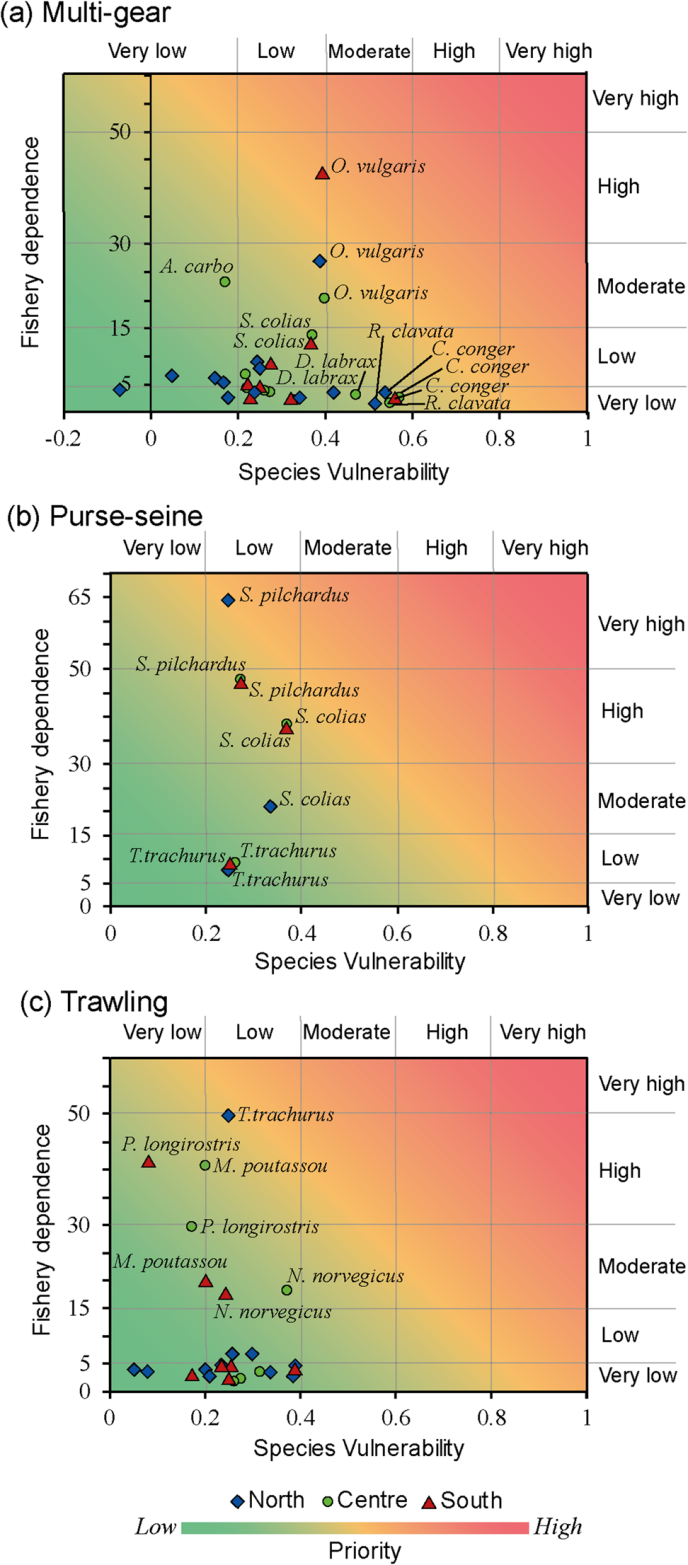
Prioritization plots of species of each fishing fleet (**a**-Multi-gear; **b**-Purse-seine; **c**-Trawling) and area (North-red; Centre-blue; South-green), considering the main species contributing to the total ecological vulnerability for the RCP 8.5 scenario (*x* axis) and considering the fisheries dependence on the species (*y* axis; average contribution of landings weight and landing economic revenue). These type of plots are adapted from ^7^. Only species that contributed to the total vulnerability of the fisheries landings weights or revenues in more than 3% are plotted and only species with higher priority are labelled. See tables of Supporting Information S7, S8 and S9 for a full list of species and classification. Green colour represents low prioritization values and red high prioritization.

In general, most of the species were in the lower-left of the prioritization plot (Fig. 6). Specifically, multi-gear fleets showed higher numbers of species with moderate vulnerability, but with low dependence on them in the three areas (Fig. 6a, Supplementary Table S7 online). However, for *O. vulgaris* in the north and centre areas the dependence was moderate and in the south the dependence was high, with more than 40% of the landings weights and revenues of multi-gear in the south being obtained from this species. In the centre area, the dependence on *A. carbo* was also classified as moderate.

In purse-seine fleets, the species targeted were classified as low vulnerability species (Supplementary Table S8 online). However, the number of species landed was low. Consequently, for of *S. pilchardus* the fishery dependence was high in the centre and south area and very high in the north (Fig. 6b). The dependence on *S. colias* was classified as high in the centre and south and *T. trachurus* was classified as low fishery dependence in the three areas (Fig. 6b).

Trawling fleet target species were mainly classified as very low or low vulnerability. On the other hand, the fishery dependence on some species was moderate or high. Specifically, in the north, the fishery had a high to very high dependence on *T. trachurus* (Fig. 6c). *M. potassou* in the centre area and *P. longirostris* in both the centre and south areas were seen as species with a high dependence from fisheries. The fishery dependence on *N. norvegicus* in the centre and south areas was moderate (Fig. 6c).

## Discussion

In many ecosystems, the high exploitation of fishing resources has resulted in the decline of long-lived species, that are usually highly sensitive to environmental changes and anthropogenic pressures^49–51^. In Portugal, there has been a decline in species that are classified as long-lived and highly sensitive, such as elasmobranchs, related to high fishing pressures^39,52^. However, no common trends in ESL were observed across areas or fishing fleets and we did not observe a general decline in the ESL between 1989 and 2015, because species with higher sensitivity were not the most landed species. Thus, their contribution to the average sensitivity was low. Also, the group of elasmobranchs composed mainly of long-lived species in many cases are under-reported in the official landings or are not reported at a species level, limiting the inclusion of these groups, such as *Raja sp.,* in the sensitivity analysis^52,53^. The reconstruction of the landings for mainland Portugal between 1938 to 2009 revealed that landings peaked in the period between 1964 and 1972 followed by a decline^42^. Hence, the years evaluated here (1989–2015) correspond to a period with declining landings and we cannot exclude the possibility that the abundance of more sensitive species were already diminished, shifting the perception of the baseline^54,55^.

Spatial differences in ESL within each fleet were probably related to the differentiated ecological assemblages off the Portuguese coast^56^, such as *S. colias,* which is more abundant in centre-south Portugal^57^, and showed greater contributions to the ESL of purse-seiners in the central and south areas compared to the north where *S. pilchardus* is the main species. Moreover, spatial differences might be a consequence of cultural and traditional specific fisheries and licensed gears, such as the case of the *A. carbo* for multi-gear fleets in the central area^58,59^. Trawling was the only fleet that presented similar trends in the ESL between areas and could be related to the lower degree of selectivity in trawling fleets^60,61^.

As expected, fluctuations in species landings partially explained the temporal changes in ecological sensitivity of the landings. Previous studies described a decline in landings of *L. caudatus* and *C. conger* in the late 1990s and an increase in *S. colias*^28,62^. The decline in moderately and highly sensitive species, such as *C. coelolepis, L. caudatus* and *C. conger,* in combination with the increase in species of low sensitivity, such as *S. colias*, explained ESL decline in the centre and south areas for multi-gear landings. The ESL in purse-seine fleets had low variability because they target pelagic short-life-span and fast-growing small and medium pelagic fishes with low sensitivity^9,63^. The increase in sensitivity in trawling fleets in the early 2000s coincided in time with significant species compositional community changes in distribution and abundance in Portugal described by Moura et al. (2020) from independent fisheries data. Other factors, such as the exploitation of new or deeper areas^37^, as well as the recovery of stocks with higher sensitivity due to management measures, may have also contributed to the increase in sensitivity of the landings in trawling fleets. The analysis of temporal changes in sensitivity of the landings allowed us to account for the life history traits of species landed and understand how this could affect the ecological vulnerability of the landings in the long term.

The ecological vulnerability of the landings is partially determined by the sensitivity of the species, but also by their exposure and adaptive capacity^20^. The EVL was similar between both climate change scenarios (RCP 4.5 and RCP 8.5), with slightly higher values for the RCP 8.5 as expected, since the reported vulnerability values of the species are higher for this scenario^39^. Differences in vulnerability between climate change scenarios were driven by the level of exposure and when combined with the information on sensitivity, adaptive capacity and landings, the weight of differences in exposure had a lower impact on the overall EVL. At spatial level, between 2010 and 2015, only multi-gear fleets presented a latitudinal gradient with an increasing EVL_(kg)_ from north to south. This latitudinal gradient was mainly driven by the increase in ecological exposure and decrease in adaptive capacity southwards. A higher exposure to environmental changes and higher vulnerability of fisheries has been described for the south of Portugal in comparison to the centre and north areas^39,62,64^. Therefore, management plans for adaptation to and mitigation of climate change should consider this regional vulnerability differences. Proposing management measures to decrease the exposure of species to climate change would be difficult, since this depends on large-scale processes^39^, but management measures to increase the adaptive capacity can be implemented at a regional level. The adaptive capacity of the species in Bueno-Pardo et al*.*^39^ was mainly calculated on indicators related to the fishing stock status and exploitation. Thus, any measure directed at decreasing overfishing and improving the sustainable management of the fisheries would increase the overall adaptive capacity of the target species and decrease the EVL^1^.

Differences in vulnerability of landing weights and revenues between fishing fleets and areas were explained by changes in species contribution to vulnerability and differences in species mean price (€·kg^−1^), highlighting the importance of considering the economic value of species for a proper understanding of the exposure of fisher communities^11,65^. Specifically, the higher vulnerability of multi-gear fleets in the south was mainly explained by *O. vulgaris,* where the fishing community highly depends on this resource^62,66^. The vulnerability of *O. vulgaris* is low to moderate^39^, but there are still important uncertainties in the direction of the effects of climate change on this species^62,67,68^. In the case of the purse-seine fleets, the differences between areas were explained by the greater dominance of *S. pilchardus* in the north and *S. colias* in the centre and south areas. Due to the sociocultural importance of sardines in Portugal^34,43^, its price is higher than the price of *S. colias* and the contribution of sardines to the EVL_(€)_ of purse-seine fleets in the central and south areas was higher. Opposite to multi-gear and purse-seine fleets, trawling fleets in the central and south areas presented the lowest EVL_(kg)_, because the main contributor was the blue whiting, which is considered a low vulnerability species with a high adaptive capacity^39^. Still, in terms of economic revenue, blue whiting has a low value and other species with higher commercial value, such as the crustaceans *P. longirostris* and *N. norvegicus,* contributed the most to EVL_(€)_. Interestingly, *P. longirostris* has increased in abundance in the Mediterranean and has shown northward expansion presenting new fishing opportunities^69,70^. Climate-related changes have been positively related to the dynamics of the stock^71^. Therefore, considering that in Portugal, the landings of this species have increased in the lasts decades^28,62^, it may contribute to increasing the resilience of trawling landings, diversifying the species targeted with the inclusion of low vulnerability species.

The vulnerability of the species is only one aspect to account for in the management of marine resources and fisheries. To increase the resilience of the fisheries, it is necessary to have an ecological-based approach for diversification of resources^37,72^. In different areas of the world, it has been observed that fishing communities that had a higher diversification of target species were able to cope better with fluctuations, not only in the landings, but also in the market prices^3,4,73^. In Portugal, the purse-seine fishing fleet presented the least species diversity. The main target species of this fishing fleet are pelagic species that tend to have higher exposure to climate change. However, the high exposure of these species is compensated with a low sensitivity which, together with measures to improve the adaptive capacity of the stocks, could lower the vulnerability of the fishery. It should be noted, however, that in the three areas considered, the fishing fleet presented a high to very high fishing dependence on sardines and a collapse of this species could have a large impact on the socio-economy of this sector^74^, as has occurred in the past due to a combination of environmental fluctuations^75,76^, poor management, and high exploitation rates^77,78^. For the purse-seine fishery, we suggest a special focus on the management of small pelagic fishes, considering the diversification of species captured, economic processing at auction (fishery valorisation) or gear/license diversification, among other measures, to increase the resilience and adaptive capacity of the purse-seine fleet landings. For example, seines could target other species with marketable interest such as anchovy, which have been increasing in purse-seine fleets recently^79^. Such changes would contribute to increasing the resilience of the seine fishery.

On the other hand, landings of the multi-gear fleet rely on a wider range of species due to the diversity of gears used. Thus, although this was found to have the highest EVL, this vulnerability is compensated with a low fishing dependence on a single species^35,62^. However, the high dependence of multi-gear fleets on *O. vulgaris* and the limited knowledge about how the population dynamics of this species might be affected by changes in the environment, should be accounted for when managing this fishery^80^. Also, considering trawling, the species portfolio was wide, but three species were classified as high fishing dependence: *T. trachurus*, *P. longirostris* and *M. potassou*. Focusing on the landing weights of *T. trachurus* and *M. potassou*, the fishing dependence was very high, but the mean auction price (€/kg) was very low. Increasing the first sell price of these species could benefit fishers’ income and potentially reduce the need for high fishing rates^81^. However, a full socio-economic assessment of the value chain of the species should be performed to avoid unintended consequences or maladaptation^82^. For example, high increases in prices for final consumers for species that are important sources of protein could have unintended consequences on communities with low wages, such as may be the case of *T. trachurus* in certain social sectors of Portugal^83^. The species prioritization plots allowed us to identify in an easy and rapid manner which are the fleets with higher dependence on more vulnerable species. This tool can be used to discuss and advice potential management strategies. But, other aspects such as conservation of key species for the ecosystem can be also combined with the criteria used.

Future fishing adaptation plans should combine information on ecological vulnerability and diversification of resources at a regional scale to ensure a successful assessment and implementation of measures, as it has been recently emphasized in a climate risk assessment at European scale^24^. The values of EVL calculated in this study express relative vulnerabilities comparing values within Portugal and only allow comparison between the study area^84^. Most estimation regardless fishing fleets rely below 0.5 vulnerability. Thus overall, the expected impact is moderate to low. The vulnerability of a species is not a static value and partially depends on adaptive capacity, which is affected by how well the resources are managed^39^. High adaptive capacity at present does necessary means that the necessary measures are being implemented to maintain this capacity in the future^85^. Considering that the stock exploitation status could change in the future, a periodic update of the vulnerability of species and ecological vulnerability of landings is required, evaluating the changes in exposure and adaptive capacity. The ecological vulnerability of the species landed, evaluated in the present study, can be considered as a measure of the ecological exposure of the fishery itself, and is a first step toward integrating ecological information within an entire socioeconomic vulnerability assessment of the fisheries.

## Supplementary Information

Supplementary Information.

## Data Availability

The datasets generated during and/or analysed during the current study are available from the corresponding author on reasonable request.

## References

[1] Sumaila UR, Tai TC (2020). End overfishing and increase the resilience of the ocean to climate change. Front. Mar. Sci..

[2] Sumaila UR (2019). Benefits of the paris agreement to ocean life, economies, and people. Sci. Adv..

[3] Beaudreau AH (2019). Thirty years of change and the future of Alaskan fisheries: Shifts in fishing participation and diversification in response to environmental, regulatory and economic pressures. Fish Fish..

[4] Finkbeiner EM (2015). The role of diversification in dynamic small-scale fisheries: Lessons from Baja California Sur. Mexico. Glob. Environ. Chang..

[5] Johnson JE (2016). Assessing and reducing vulnerability to climate change: Moving from theory to practical decision-support. Mar. Policy.

[6] IPCC. Climate Change 2007: Synthesis Report. Contribution of working groups I, II and III to the fourth assessment report of the intergovernmental panel on climate change. (2007).

[7] Johnson JE, Welch DJ (2016). Climate change implications for Torres Strait fisheries: Assessing vulnerability to inform adaptation. Clim. Change.

[8] IPCC. Annex I: Glossary. in IPCC special report on the ocean and cryosphere in a changing climate e [H.-O. Pörtner, D.C. Roberts, V. Masson-Delmotte, P. Zhai, M. Tignor, E. Poloczanska, K. Mintenbeck, A. Alegría, M. Nicolai, A. Okem, J. Petzold, B. Rama, N.M. Weyer (eds.)] 677–702 (Cambridge University Press, 2019). 10.1017/9781009157964.010

[9] Cheung WWL, Watson R, Morato T, Pitcher TJ, Pauly D (2007). Intrinsic vulnerability in the global fish catch. Mar. Ecol. Prog. Ser..

[10] Pauly D, Christensen V, Dalsgaard J, Froese R, Torres F (1998). Fishing down marine food webs. Science.

[11] Lam VWY, Cheung WWL, Reygondeau G, Rashid Sumaila U (2016). Projected change in global fisheries revenues under climate change. Sci. Rep..

[12] Heck N (2020). Fisheries at risk: Vulnerability of fisheries to climate change.

[13] Allison EH (2009). Vulnerability of national economies to the impacts of climate change on fisheries. Fish Fish..

[14] DuFour MR (2015). Portfolio theory as a management tool to guide conservation and restoration of multi-stock fish populations. Ecosphere.

[15] Kasperski S, Holland DS (2013). Income diversification and risk for fishermen. Proc. Natl. Acad. Sci. U. S. A..

[16] Bahri, T. et al. Adaptive management of fisheries in response to climate change. FAO Fisheries and Aquaculture Technical Paper **667**, (FAO, 2021).

[17] Barker MJ, Schluessel V (2005). Managing global shark fisheries: Suggestions for prioritizing management strategies. Aquat. Conserv. Mar. Freshw. Ecosyst..

[18] Fletcher WJF, Fletcher WJ (2005). The application of qualitative risk assessment methodology to prioritize issues for fisheries management. ICES J. Mar. Sci..

[19] Cheung WWL (2018). The future of fishes and fisheries in the changing oceans. J. Fish Biol..

[20] Cinner JE (2013). Evaluating social and ecological vulnerability of coral reef fisheries to climate change. PLoS ONE.

[21] Colburn LL (2016). Indicators of climate change and social vulnerability in fishing dependent communities along the Eastern and Gulf Coasts of the United States. Mar. Policy.

[22] Pinnegar JK (2019). Assessing vulnerability and adaptive capacity of the fisheries sector in Dominica: Long-term climate change and catastrophic hurricanes. ICES J. Mar. Sci..

[23] Aragão GM (2021). The importance of regional differences in vulnerability to climate change for demersal fisheries. ICES J. Mar. Sci..

[24] Payne MR, Kudahl M, Engelhard GH, Peck MA, Pinnegar JK (2021). Climate risk to European fisheries and coastal communities. Proc. Natl. Acad. Sci. U. S. A..

[25] Baptista V, Silva PL, Relvas P, Teodósio MA, Leitão F (2018). Sea surface temperature variability along the Portuguese coast since 1950. Int. J. Climatol..

[26] Leitão F (2019). (2019) A 60-year time series analyses of the upwelling along the Portuguese coast. Water.

[27] Leitão F, Relvas P, Cánovas F, Baptista V, Teodósio A (2018). Northerly wind trends along the Portuguese marine coast since 1950. Theor. Appl. Climatol..

[28] Bueno-Pardo J (2020). Trends and drivers of marine fish landings in Portugal since its entrance in the European Union. ICES J. Mar. Sci..

[29] Leitão F, Maharaj RR, Vieira VMNCS, Teodósio A, Cheung WWL (2018). The effect of regional sea surface temperature rise on fisheries along the Portuguese Iberian Atlantic coast. Aquat. Conserv. Mar. Freshw. Ecosyst..

[30] Leitão F, Alms V, Erzini K (2014). A multi-model approach to evaluate the role of environmental variability and fishing pressure in sardine fisheries. J. Mar. Syst..

[31] Ullah H, Leitão F, Baptista V, Chícharo L (2012). An analysis of the impacts of climatic variability and hydrology on the coastal fisheries, Engraulis encrasicolus and Sepia officinalis, of Portugal. Ecohydrol. Hydrobiol..

[32] EUMOFA. The EU Fish Market - Highlights the EU in the world market supply consumption import-export landings in the EU aquaculture (2021) 10.2771/563899

[33] DGPM. Relatório de Monitorização da Estratégia Nacional para o Mar 2013–2020, Documento de Suporte às Políticas do Mar. (2020).

[34] Almeida C, Karadzic V, Vaz S (2015). The seafood market in Portugal: Driving forces and consequences. Mar. Policy.

[35] Pita, C. & Gaspar, M. (2020) Small-Scale Fisheries in Portugal: Current Situation, Challenges and Opportunities for the Future. In Small-Scale Fisheries in Europe: Status, Resilience and Governance. Springer, Cham 283–30510.1007/978-3-030-37371-9_14

[36] Baeta F, José Costa M, Cabral H (2009). Changes in the trophic level of Portuguese landings and fish market price variation in the last decades. Fish. Res..

[37] Leitão F (2015). Landing profiles of Portuguese fisheries: Assessing the state of stocks. Fish. Manag. Ecol..

[38] Quentin Grafton R (2010). Adaptation to climate change in marine capture fisheries. Mar. Policy.

[39] Bueno-Pardo J (2021). Climate change vulnerability assessment of the main marine commercial fish and invertebrates of Portugal. Sci. Rep..

[40] Szynaka MJ, Erzini K, Gonçalves JMS, Campos A (2021). Identifying métiers using landings profiles: An octopus-driven multi-gear coastal fleet. J. Mar. Sci. Eng..

[41] Gamito R, Teixeira CM, Costa MJ, Cabral HN (2013). Climate-induced changes in fish landings of different fleet components of Portuguese fisheries. Reg. Environ. Chang..

[42] Leitão F, Baptista V, Zeller D, Erzini K (2014). Reconstructed catches and trends for mainland Portugal fisheries between 1938 and 2009: Implications for sustainability, domestic fish supply and imports. Fish. Res..

[43] Teixeira CM (2014). Trends in landings of fish species potentially affected by climate change in Portuguese fisheries. Reg. Environ. Chang..

[44] Wickham H (2016). ggplot2: Elegant graphics for data analysis.

[45] R Core Team. R: A language and environment for statistical computing. R Foundation for Statistical Computing, Vienna, Austria 3–900051–07–0 (2020).

[46] Zuur AF, Fryer RJ, Jolliffe IT, Dekker R, Beukema JJ (2003). Estimating common trends in multivariate time series using dynamic factor analysis. Environmetrics.

[47] Zuur, A. F., Ieno, E. N. & Smith, G. M. (2007) Analysing Ecological Data. 10.1007/978-0-387-45972-1

[48] Anderson, M., Gorley, R. & Clarke, K. PERMANOVA for PRIMER: Guide to software and statistical methods. (PRIMER-E Ltd., 2008).

[49] Heppell, S. S., Heppell, S. a, Read, A. J. & Crowder, L. B. Effects of fishing on long-lived marine organisms. In Marine conservation biology: The science of maintaining the sea’s biodiversity (eds. Norse, E. & Crowder, L.) 211–231 (Island Press, 2005).

[50] Maynou F (2011). Estimating trends of population decline in long-lived marine species in the Mediterranean sea based on fishers’ perceptions. PLoS ONE.

[51] Rolland V, Barbraud C, Weimerskirch H (2008). Combined effects of fisheries and climate on a migratory long-lived marine predator. J. Appl. Ecol..

[52] Alves LMF, Correia JPS, Lemos MFL, Novais SC, Cabral H (2020). Assessment of trends in the Portuguese elasmobranch commercial landings over three decades (1986–2017). Fish. Res..

[53] Correia JP, Morgado F, Erzini K, Soares AMVM (2016). Elasmobranch landings for the Portuguese commercial fishery from 1986 to 2009. Arquipel. Life Mar. Sci..

[54] Pauly D (1995). Anecdotes and the shifting baseline syndrome of fisheries. Trends Ecol. Evol..

[55] Pinnegar JK, Engelhard GH (2008). The ‘shifting baseline’ phenomenon: A global perspective. Rev. Fish Biol. Fish..

[56] Moura T (2020). Assessing spatio-temporal changes in marine communities along the Portuguese continental shelf and upper slope based on 25 years of bottom trawl surveys. Mar. Environ. Res..

[57] Martins MM, Skagen D, Marques V, Zwolinski J, Silva A (2013). Changes in the abundance and spatial distribution of the Atlantic chub mackerel (*Scomber colias*) in the pelagic ecosystem and fisheries off Portugal. Sci. Mar..

[58] Bordalo-Machado P, Figueiredo I (2009). The fishery for black scabbardfish (Aphanopus carbo Lowe, 1839) in the Portuguese continental slope. Rev. Fish Biol. Fish..

[59] Gordo LS (2009). Black scabbardfish (Aphanopus carbo Lowe, 1839) in the southern Northeast Atlantic: Considerations on its fishery. Sci. Mar..

[60] Campos A, Fonseca P, Fonseca T, Parente J (2007). Definition of fleet components in the Portuguese bottom trawl fishery. Fish. Res..

[61] Bueno-Pardo J (2017). Deep-sea crustacean trawling fisheries in Portugal: Quantification of effort and assessment of landings per unit effort using a Vessel Monitoring System (VMS). Sci. Rep..

[62] Gamito R, Pita C, Teixeira C, Costa MJ, Cabral HN (2016). Trends in landings and vulnerability to climate change in different fleet components in the Portuguese coast. Fish. Res..

[63] García-Seoane E, Marques V, Silva A, Angélico MM (2019). Spatial and temporal variation in pelagic community of the western and southern Iberian Atlantic waters. Estuar. Coast. Shelf Sci..

[64] Vinagre C, Duarte F, Cabral H, Jose M (2011). Impact of climate warming upon the fish assemblages of the Portuguese coast under different scenarios. Reg. Environ. Change.

[65] Goulart P, Veiga FJ, Grilo C (2018). The evolution of fisheries in Portugal: A methodological reappraisal with insights from economics. Fish. Res..

[66] Pita, C., Pereira, J., Lourenço, S., Sonderblohm, C. & Pierce, G. J. (2015) The Traditional Small-Scale Octopus Fishery in Portugal: Framing Its Governability. 117–132. 10.1007/978-3-319-17034-3_7

[67] Pita C (2021). Fisheries for common octopus in Europe: Socioeconomic importance and management. Fish. Res..

[68] Moreno A (2014). Essential habitats for pre-recruit Octopus vulgaris along the Portuguese coast. Fish. Res..

[69] Sbrana M (2019). Spatiotemporal abundance pattern of deep-water rose shrimp, parapenaeus longirostris, and Norway lobster, nephrops norvegicus, in european mediterranean waters. Sci. Mar..

[70] Quattrocchi F, Fiorentino F, Lauria V, Garofalo G (2020). The increasing temperature as driving force for spatial distribution patterns of Parapenaeus longirostris (Lucas 1846) in the Strait of Sicily (Central Mediterranean Sea). J. Sea Res..

[71] Colloca F, Mastrantonio G, Lasinio GJ, Ligas A, Sartor P (2014). Parapenaeus longirostris (Lucas, 1846) an early warning indicator species of global warming in the central Mediterranean Sea. J. Mar. Syst..

[72] Woods, P. J. *et al.* (2021) A review of adaptation options in fisheries management to support resilience and transition under socio-ecological change. ICES J. Mar. Sci. **fsab146**

[73] Gonzalez-Mon B (2021). Spatial diversification as a mechanism to adapt to environmental changes in small-scale fisheries. Environ. Sci. Policy.

[74] Garza-Gil MD, Torralba-Cano J, Varela-Lafuente MM (2011). Evaluating the economic effects of climate change on the European sardine fishery. Reg. Environ. Chang..

[75] Borges MF, Santos AMP, Crato N, Mendes H, Mota B (2003). Sardine regime shifts off Portugal: A time series analysis of catches and wind conditions. Sci. Mar..

[76] Garrido S (2017). Temperature and food-mediated variability of European Atlantic sardine recruitment. Prog. Oceanogr..

[77] ICES. Report of the working group on southern horse mackerel, anchovy and sardine (WGHANSA). (2018).

[78] Szalaj D (2021). Food-web dynamics in the Portuguese continental shelf ecosystem between 1986 and 2017: Unravelling drivers of sardine decline. Estuar. Coast. Shelf Sci..

[79] Feijó D (2019). Catch and yield trends of the Portuguese purse seine fishery (2006–2018). Front. Mar. Sci..

[80] Schickele A, Francour P, Raybaud V (2021). European cephalopods distribution under climate-change scenarios. Sci. Rep..

[81] Purcell SW, Crona BI, Lalavanua W, Eriksson H (2017). Distribution of economic returns in small-scale fisheries for international markets: A value-chain analysis. Mar. Policy.

[82] Thiao D, Leport J, Ndiaye B, Mbaye A (2018). Need for adaptive solutions to food vulnerability induced by fish scarcity and unaffordability in Senegal. Aquat. Living Resour..

[83] Education A, Variability H (2016). Cardoso, C., Lourenço, H., Costa, S., Gonçalves, S. & Leonor Nunes, M. Survey Into the Seafood Consumption Preferences and Patterns in the Portuguese Population. J. Food Prod. Mark..

[84] Holsten A, Kropp JP (2012). An integrated and transferable climate change vulnerability assessment for regional application. Nat. Hazards.

[85] Umweltbundesamt guidelines for climate impact and vulnerability assessments recommendations of the interministerial working group on adaptation to climate change of the German federal government for our environment.

