# Supplementary material for: Ecological sensitivity and vulnerability of fishing fleet landings to climate change across regions
Source: Sci Rep. 2022 Oct 17;12:17360. doi: 10.1038/s41598-022-21284-3 (PMC9576743; doi:10.1038/s41598-022-21284-3)
Supplement: Supplementary file 1 — Supplementary Information. [file 41598_2022_21284_MOESM1_ESM.docx]

**SUPPLEMENTARY INFORMATION**

**Ecological Sensitivity and Vulnerability of fishing fleet landings to climate change across regions**

Marta Albo-Puigserver^1,2^, Juan Bueno-Pardo^3^, Miguel Pinto^1^, João N. Monteiro^1^, Andreia Ovelheiro^1^, Maria A. Teodósio^1^, Francisco Leitão^1^

^1^ Centro de Ciências do Mar (CCMAR), Universidade do Algarve, Campus de Gambelas, 8005‑139 Faro, Portugal.

^2^Centro Oceanográfico de Baleares, Instituto Español de Oceanografía (IEO-CSIC), Ecosystem Oceanography Group, 07015 Palma, Spain

^3^Centro de Investigación Mariña, Universidade de Vigo, Future Oceans Lab, Lagoas-Marcosende, 36310 Vigo, Spain.

| **Appendix Supplementary Information 1:** Tables | |
| --- | --- |
| **Table** | **Page** |
| **Table S1.** (A) Values of Akaike’s information criterion (AIC) for DFA models with a variable number of common trends (M= 1 to 4) and noise. (B) Canonical correlations between response variables (sensitivity time series) and DFA common trends. Correlations higher than 0.5 are indicated in bold. | 3 |
| **Table S2**. SIMPER analysis results of multi-gear fleet vulnerability between areas in terms of landings weight (A) and landings economic revenue (B). Only species that made up to 50% of the cumulative contribution to dissimilarity in vulnerability are reported. | 4 |
| **Table S3**. SIMPER analysis results of purse-seine fleet vulnerability between areas in terms of landings weight (A) and landings economic revenue (B). Only species that made up to 50% of the cumulative contribution to dissimilarity in vulnerability are reported. | 6 |
| **Table S4**. SIMPER analysis results of trawl fleet vulnerability between areas in terms of landings weight (A) and economic revenue (B). Only species that made up to 50% of the cumulative contribution to dissimilarity in vulnerability are reported. | 7 |
| **Table S5**. Kruskal-Wallis test and Wilcoxon rank sum test for each component of vulnerability of landings weight (sensitivity, exposure and adaptive capacity) comparing between areas (North, Centre and South) and fishing fleets (Multi-gear; *Multi*, Purse-seine; *Seine*, Trawling; *Trawl*).* significant p-value (p<0.05). | 8 |
| **Table S6**. Kruskal-Wallis test and Wilcoxon rank sum test for each component of vulnerability of landings revenue (sensitivity, exposure and adaptive capacity) comparing between areas (North, Centre and South) and fishing fleets (Multi-gear; *Multi*, Purse-seine; *Seine*, Trawling; *Trawl*).* significant p-value (p<0.05). | 9 |
| **Table S7.** Muli-gear fishery dependence and species vulnerability for each species landed. Only species that had a contribution higher than 3% to the total vulnerability (Vul) of the landings and or to the total landings weight (W) and revenue (Rev) were included in the analysis. Mean values correspond to the average values of landing weight and landing revenue proportion. The categorization in very low (dark green), low (light green), moderate (yellow) and high (light red) are indicated according to the classification proposed in the section 2.3. | 10 |
| **Table S8.** Purse-seine fishery dependence and species vulnerability for each species landed. Only species that had a contribution higher than 3% to the total vulnerability (Vul) of the landings and or to the total landings weight (W) and revenue (Rev) were included in the analysis. Mean values correspond to the average values of landing weight and landing revenue proportion. The categorization in very low (dark green), low (light green), moderate (yellow), high (light red) and very high (red) are indicated according to the classification proposed in the section 2.3. | 11 |
| **Table S9.** Trawling fishery dependence and species vulnerability for each species landed. Only species that had a contribution higher than 3% to the total vulnerability (Vul) of the landings and or to the total landings weight (W) and revenue (Rev) were included in the analysis. Mean values correspond to the average values of landing weight and landing revenue proportion. The categorization in very low (dark green), low (light green), moderate (yellow) and high (light red) are indicated according to the classification proposed in the section 2.3. | 12 |

| **Appendix Supporting Information 2:** Figures | |
| --- | --- |
| **Figure** | **Page** |
| **Fig. S1**. Multi-gear fishery sensitivity contribution (A, B, C) and landings (thousand tons; D, E, F) by year for the main species that contributed to the total sensitivity (>3%) in the north (A,D), centre (B,E) and south (C, F) area. | 13 |
| **Fig. S2**. Purse-seine fishery sensitivity contribution (A, B, C) and landings (thousand tons; D, E, F) by year for the main species that contributed to the total sensitivity (>3%) in the north (A,D), centre (B,E) and south (C, F) area. | 14 |
| **Fig. S3**. Trawling fishery sensitivity contribution (A, B, C) and landings (thousand tons; D, E, F) by year for the main species that contributed to the total sensitivity (>3%) in the north (A,D), centre (B,E) and south (C, F) area. | 15 |
| **Fig. S4**. Ecological vulnerability of the landings weight (a) and economic revenue (b) and ecological exposure of the landings weight (c) and ecoomic revenue (d) by gear type and area calculated for the average period 2010-2015 and RCP 4.5. Each gear type is represented with a different colour. | 16 |

| **Supplementary Table S1.** (A) Values of Akaike’s information criterion (AIC) for DFA models with a variable number of common trends (M= 1 to 4) and noise. (B) Canonical correlations between response variables (sensitivity time series) and DFA common trends. Correlations higher than 0.5 are indicated in bold. | | | |
| --- | --- | --- | --- |
| 1. **Model** | **AIC** | | |
| 1 trend +noise | 595.581 | | |
| 2 trends + noise | 572.809 | | |
| **3 trends + noise** | **554.480** | | |
| 4 trends + noise | 560.601 | | |
| 1. **Time-series** | **Trend 1** | **Trend 2** | **Trend 3** |
| Multi-gear North | **0.969** | -0.065 | 0.174 |
| Multi-gear Central | 0.086 | **-0.977** | 0.361 |
| Multi-gear South | **0.500** | -0.112 | **0.861** |
| Purse-seine North | 0.321 | 0.480 | 0.311 |
| Purse-seine Central | 0.115 | -0.237 | **0.602** |
| Purse-seine South | 0.294 | 0.025 | 0.344 |
| Trawling North | 0.000 | **0.755** | -0.093 |
| Trawling Central | -0.004 | **0.821** | **-0.529** |
| Trawling South | -0.284 | **0.579** | 0.066 |

| **Supplementary Table S2**. SIMPER analysis results of multi-gear fleet vulnerability between areas in terms of landings weight (A) and landings economic revenue (B). Only species that made up to 50% of the cumulative contribution to dissimilarity in vulnerability are reported. | | | | | |
| --- | --- | --- | --- | --- | --- |
| Species | Abund.1 | Abund.2 | Average Dissimilarity | Contribution dissimilarity (%) | Cumulative contribution (%) |
| 1. **Vulnerability Landings Weight** | | | | | |
| **North-Central** (*Average dissimilarity = 31.10*) | | | | | |
| *Scomber colias* | 0.11 | 0.30 | 4.35 | 13.97 | 13.97 |
| *Cerastoderma edule* | 0.11 | 0.00 | 2.37 | 7.63 | 21.60 |
| *Aphanopus carbo* | 0.09 | 0.20 | 2.36 | 7.58 | 29.18 |
| *Sardina pilchardus* | 0.15 | 0.11 | 2.05 | 6.58 | 35.76 |
| *Callista chione* | 0 | 0.09 | 1.98 | 6.37 | 42.13 |
| *Merluccius merluccius* | 0.14 | 0.08 | 1.36 | 4.37 | 46.50 |
| *Octopus vulgaris* | 0.29 | 0.25 | 1.23 | 3.94 | 50.44 |
| **North-South** (*Average dissimilarity = 34.26*) | | | | | |
| *Scomber colias* | 0.11 | 0.27 | 3.63 | 10.61 | 10.61 |
| *Cerastoderma edule* | 0.11 | 0 | 2.44 | 7.12 | 17.72 |
| *Sardina pilchardus* | 0.15 | 0.15 | 2.32 | 6.78 | 24.51 |
| *Octopus vulgaris* | 0.29 | 0.36 | 2.29 | 6.69 | 31.19 |
| *Donax* spp. | 0 | 0.08 | 1.84 | 5.36 | 36.55 |
| *Aphanopus carbo* | 0.09 | 0.01 | 1.83 | 5.36 | 41.91 |
| *Merluccius merluccius* | 0.14 | 0.07 | 1.53 | 4.48 | 46.38 |
| *Trachurus trachurus* | 0.16 | 0.11 | 1.23 | 3.59 | 49.98 |
| *Diplodus vulgaris* | 0.02 | 0.07 | 1.09 | 3.19 | 53.17 |
| **Centr-South** (*Average dissimilarity = 27.33*) | | | | | |
| *Aphanopus carbo* | 0.20 | 0.01 | 4.14 | 15.15 | 15.15 |
| *Octopus vulgaris* | 0.25 | 0.36 | 2.74 | 10.03 | 25.18 |
| *Scomber colias* | 0.30 | 0.27 | 2.04 | 7.47 | 32.66 |
| *Callista chione* | 0.09 | 0.00 | 1.98 | 7.24 | 39.90 |
| *Sardina pilchardus* | 0.11 | 0.15 | 1.72 | 6.31 | 46.21 |
| *Prionace glauca* | 0.07 | 0.02 | 1.04 | 3.79 | 50.00 |
| 1. **Vulnerability Landings Revenue** | | | | | |
| **Economic Revenue North-Centre** (*Average dissimilarity = 22.93*) | | | | | |
| *Aphanopus carbo* | 0.10 | 0.20 | 2.14 | 9.33 | 9.33 |
| *Cerastoderma edule* | 0.06 | 0.00 | 1.35 | 5.90 | 15.22 |
| *Sepia officinalis* | 0.07 | 0.13 | 1.25 | 5.44 | 20.67 |
| *Merluccius merluccius* | 0.14 | 0.08 | 1.14 | 5.39 | 26.05 |
| *Callista chione* | 0.00 | 0.05 | 1.14 | 4.98 | 31.03 |
| *Sardina pilchardus* | 0.09 | 0.08 | 1.12 | 4.90 | 35.93 |
| *Scomber colias* | 0.05 | 0.09 | 0.98 | 4.25 | 40.18 |
| *Spisula solida* | 0.05 | 0.01 | 0.90 | 3.91 | 44.08 |
| *Sparus aurata* | 0.07 | 0.10 | 0.80 | 3.47 | 47.55 |
| *Octopus vulgaris* | 0.35 | 0.32 | 0.78 | 3.39 | 50.95 |
| **North-South** (*Average dissimilarity =29.35*) | | | | | |
| *Aphanopus carbo* | 0.10 | 0.01 | 1.91 | 6.52 | 6.52 |
| *Octopus vulgaris* | 0.35 | 0.44 | 1.90 | 6.48 | 13.01 |
| *Merluccius merluccius* | 0.14 | 0.07 | 1.60 | 5.45 | 18.45 |
| *Donax* spp. | 0.00 | 0.08 | 1.58 | 5.39 | 23.84 |
| *Sardina pilchardus* | 0.09 | 0.12 | 1.39 | 4.74 | 28.58 |
| *Cerastoderma edule* | 0.06 | 0.00 | 1.38 | 4.70 | 33.28 |
| *Dicentrarchus labrax* | 0.15 | 0.10 | 1.10 | 3.76 | 37.04 |
| *Scomber colias* | 0.05 | 0.09 | 1.01 | 3.43 | 40.47 |
| *Mullus surmuletus* | 0.05 | 0.09 | 0.95 | 3.24 | 43.71 |
| *Sepia officinalis* | 0.07 | 0.12 | 0.88 | 3.00 | 46.71 |
| **Centre-South** (*Average dissimilarity =25.94*) | | | | | |
| *Aphanopus carbo* | 0.20 | 0.01 | 4.05 | 15.60 | 15.60 |
| *Octopus vulgaris* | 0.32 | 0.44 | 2.58 | 9.95 | 25.54 |
| *Dicentrarchus labrax* | 0.15 | 0.10 | 1.19 | 4.59 | 30.13 |
| *Callista chione* | 0.05 | 0.00 | 1.15 | 4.43 | 34.56 |
| *Sardina pilchardus* | 0.08 | 0.12 | 1.13 | 4.36 | 38.93 |
| *Solea solea* | 0.12 | 0.07 | 1.06 | 4.10 | 43.03 |
| *Lophius* spp*.* | 0.06 | 0.10 | 0.89 | 3.45 | 46.48 |
| *Donax* spp. | 0.04 | 0.08 | 0.82 | 3.17 | 49.65 |

| **Supplementary Table S3**. SIMPER analysis results of purse-seine fleet vulnerability between areas in terms of landings weight (A) and landings economic revenue (B). Only species that made up to 50% of the cumulative contribution to dissimilarity in vulnerability are reported. | | | | | | | | | | |  |
| --- | --- | --- | --- | --- | --- | --- | --- | --- | --- | --- | --- |
| Species | Abund.1 | | Abund.2 | | Average Dissimilarity | | Contribution dissimilarity (%) | | Cumulative contribution (%) | |  |
| 1. **Vulnerability Landings Weight** | | | | | | | | | | |  |
| **North-Central** (*Average dissimilarity = 21.19*) | | | | | | | | | | |  |
| *Scomber colias* | 0.32 | | 0.45 | | 6.47 | | 30.54 | | 30.56 | |  |
| *Sardina pilchardus* | 0.37 | | 0.29 | | 4.56 | | 21.53 | | 52.08 | |  |
| **North-South** (*Average dissimilarity = 22.37*) | | | | | | | | | | |  |
| *Scomber colias* | 0.32 | | 0.45 | | 6.31 | | 28.21 | | 28.21 | |  |
| *Sardina pilchardus* | 0.37 | | 0.29 | | 4.36 | | 19.51 | | 47.73 | |  |
| *Trachurus trachurus* | 0.14 | | 0.12 | | 2.04 | | 9.12 | | 56.85 | |  |
| **Centre-South** (*Average dissimilarity = 14.59*) | | | | | | | | | | |  |
| *Sardina pilchardus* | 0.29 | | 0.29 | | 3.47 | | 23.80 | | 23.80 | |  |
| *Scomber colias* | 0.45 | | 0.45 | | 2.40 | | 16.44 | | 40.24 | |  |
| *Trachurus trachurus* | 0.14 | | 0.12 | | 1.99 | | 13.65 | | 53.89 | |  |
| 1. **Vulnerability Landings Revenue** | | | | | | | | | | | |
| **North-Central** (*Average dissimilarity = 19.39*) | | | | | | | | | | | |
| *Scomber colias* | | 0.19 | | 0.27 | | 3.43 | | 17.70 | | 17.70 | |
| *Engraulis encrasicolus* | | 0.07 | | 0.02 | | 2.41 | | 12.44 | | 30.14 | |
| *Pagellus acarne* | | 0.02 | | 0.07 | | 2.00 | | 10.29 | | 40.44 | |
| *Trachurus trachurus* | | 0.13 | | 0.16 | | 1.38 | | 7.11 | | 47.55 | |
| *Diplodus vulgaris* | | 0.02 | | 0.05 | | 1.36 | | 7.01 | | 54.56 | |
| **North-South** (*Average dissimilarity = 21.02*) | | | | | | | | | | | |
| *Scomber colias* | | 0.19 | | 0.25 | | 2.51 | | 11.97 | | 11.97 | |
| *Diplodus vulgaris* | | 0.02 | | 0.06 | | 1.74 | | 8.29 | | 20.26 | |
| *Lithognathus mormyrus* | | 0.00 | | 0.04 | | 1.56 | | 7.42 | | 27.68 | |
| *Engraulis encrasicolus* | | 0.07 | | 0.04 | | 1.54 | | 7.35 | | 35.03 | |
| *Pagellus acarne* | | 0.02 | | 0.06 | | 1.52 | | 7.23 | | 42.26 | |
| *Diplodus sardus* | | 0.03 | | 0.06 | | 1.44 | | 6.86 | | 49.12 | |
| *Trachurus trachurus* | | 0.13 | | 0.16 | | 1.33 | | 6.34 | | 55.46 | |
| **Centre-South** (*Average dissimilarity = 14.76*) | | | | | | | | | | | |
| *Scomber colias* | | 0.27 | | 0.25 | | 1.58 | | 10.69 | | 10.69 | |
| *Lithognathus mormyrus* | | 0.01 | | 0.04 | | 1.22 | | 8.27 | | 18.96 | |
| *Engraulis encrasicolus* | | 0.02 | | 0.04 | | 1.19 | | 8.03 | | 26.99 | |
| *Sardina pilchardus* | | 0.41 | | 0.41 | | 1.16 | | 7.85 | | 34.85 | |
| *Trachurus trachurus* | | 0.16 | | 0.16 | | 0.97 | | 6.56 | | 41.41 | |
| *Trachurus picturatus* | | 0.05 | | 0.07 | | 0.91 | | 6.16 | | 47.57 | |
| *Pagellus acarne* | | 0.07 | | 0.06 | | 0.87 | | 5.92 | | 53.50 | |

| **Supplementary Table S4**. SIMPER analysis results of trawl fleet vulnerability between areas in terms of landings weight (A) and economic revenue (B). Only species that made up to 50% of the cumulative contribution to dissimilarity in vulnerability are reported. | | | | | |
| --- | --- | --- | --- | --- | --- |
| Species | Abund.1 | Abund.2 | Average Dissimilarity | Contribution dissimilarity (%) | Cumulative contribution (%) |
| 1. **Vulnerability Landings Weight** | | | | | |
| **North-Centre** (*Average dissimilarity = 59.15*) | | | | | |
| *Trachurus trachurus* | 0.37 | 0.08 | 10.07 | 17.03 | 17.03 |
| *Micromesistius poutassou* | 0.10 | 0.38 | 9.62 | 16.27 | 33.30 |
| *Nephrops norvegicus* | 0.02 | 0.15 | 4.55 | 7.69 | 40.98 |
| *Trachurus picturatus* | 0.17 | 0.05 | 4.38 | 7.41 | 48.39 |
| *Scomber colias* | 0.13 | 0.01 | 3.97 | 6.71 | 55.10 |
| **North-South** (*Average dissimilarity = 50.77*) | | | | | |
| *Trachurus trachurus* | 0.37 | 0.09 | 8.35 | 16.45 | 16.45 |
| *Parapenaeus longirostris* | 0.03 | 0.20 | 5.28 | 10.40 | 26.85 |
| *Nephrops norvegicus* | 0.02 | 0.19 | 5.12 | 10.08 | 36.93 |
| *Micromesistius poutassou* | 0.10 | 0.26 | 4.79 | 9.44 | 46.37 |
| *Trachurus picturatus* | 0.17 | 0.02 | 4.53 | 8.92 | 55.29 |
| **Centre-South** (*Average dissimilarity = 32.01*) | | | | | |
| *Micromesistius poutassou* | 0.38 | 0.26 | 4.10 | 12.81 | 12.81 |
| *Octopus vulgaris* | 0.03 | 0.13 | 3.58 | 11.20 | 24.01 |
| *Parapenaeus longirostris* | 0.13 | 0.20 | 2.63 | 8.23 | 32.24 |
| *Sepia officinalis* | 0.01 | 0.08 | 2.47 | 7.72 | 39.96 |
| *Merluccius merluccius* | 0.08 | 0.13 | 1.74 | 5.44 | 45.40 |
| *Lophius* spp*.* | 0.08 | 0.12 | 1.54 | 4.81 | 50.21 |
| 1. **Vulnerability Landings Revenue** | | | | | |
| **North-Centre** (*Average dissimilarity = 61.90*) | | | | | |
| *Trachurus trachurus* | 0.33 | 0.04 | 9.27 | 14.97 | 14.97 |
| *Nephrops norvegicus* | 0.05 | 0.33 | 8.93 | 14.43 | 29.40 |
| *Parapenaeus longirostris* | 0.07 | 0.29 | 6.84 | 11.04 | 40.45 |
| *Loligo vulgaris* | 0.14 | 0.02 | 3.91 | 6.31 | 46.76 |
| *Octopus vulgaris* | 0.15 | 0.03 | 3.70 | 5.98 | 52.74 |
| **North-South** (*Average dissimilarity = 54.37*) | | | | | |
| *Trachurus trachurus* | 0.33 | 0.04 | 9.01 | 16.57 | 16.57 |
| *Nephrops norvegicus* | 0.05 | 0.30 | 7.45 | 13.71 | 30.28 |
| *Parapenaeus longirostris* | 0.07 | 0.31 | 7.07 | 13.00 | 43.29 |
| *Loligo vulgaris* | 0.14 | 0.05 | 2.97 | 5.46 | 48.75 |
| *Pagellus acarne* | 0.12 | 0.04 | 2.61 | 4.80 | 53.55 |
| **Centre-South** (*Average dissimilarity = 21.73*) | | | | | |
| *Octopus vulgaris* | 0.03 | 0.11 | 2.85 | 13.10 | 13.10 |
| *Micromesistius poutassou* | 0.14 | 0.07 | 2.63 | 12.11 | 25.21 |
| *Sepia officinalis* | 0.01 | 0.07 | 2.08 | 9.58 | 34.79 |
| *Nephrops norvegicus* | 0.33 | 0.30 | 1.69 | 7.80 | 42.58 |
| *Lophius* spp. | 0.11 | 0.11 | 1.59 | 7.33 | 49.91 |
| *Merluccius merluccius* | 0.07 | 0.08 | 1.02 | 4.71 | 54.62 |

| **Supplementary Table S5**. Kruskal-Wallis test and Wilcoxon rank sum test for each component of vulnerability of landings weight (sensitivity, exposure and adaptive capacity) comparing between areas (North, Centre and South) and fishing fleets (Multi-gear; *Multi*, Purse-seine; *Seine*, Trawling; *Trawl*).* significant p-value (p<0.05). | | | | | | | |
| --- | --- | --- | --- | --- | --- | --- | --- |
| **Landings Weight** | | **Kruskal-Wallis test** | | | **Wilcoxon test** | | |
| *Fishing fleet comparisons* | | df | H | p-value | Multi-Seine | Multi-Trawl | Seine-Trawl |
| Sensitivity | North | 2 | 11.474 | 0.003* | 0.003* | 0.699 | 0.003* |
|  | Centre | 2 | 13.661 | 0.001* | 0.003* | 0.026* | 0.003* |
|  | South | 2 | 14.749 | <0.001* | 0.003* | 0.004* | 0.003* |
| Exposure | North | 2 | 12.538 | 0.002* | 0.003* | 0.132 | 0.003* |
|  | Centre | 2 | 15.158 | <0.001* | 0.002* | 0.002* | 0.002* |
|  | South | 2 | 15.158 | <0.001* | 0.002* | 0.002* | 0.002* |
| Adaptive capacity | North | 2 | 11.942 | 0.003* | 0.003* | 0.310 | 0.003* |
|  | Centre | 2 | 15.158 | <0.001* | 0.002* | 0.002* | 0.002* |
|  | South | 2 | 15.158 | <0.001* | 0.002* | 0.002* | 0.002* |
| *Area comparisons* | | df | H | p-value | North-Centre | North-South | Centre-South |
| Sensitivity | Muti-gear | 2 | 10.526 | 0.005* | 0.065 | 0.065 | 0.007* |
|  | Purse-seine | 2 | 0.666 | 0.716 |  |  |  |
|  | Trawling | 2 | 10.211 | 0.006* | 0.007* | 0.394 | 0.023* |
| Exposure | Muti-gear | 2 | 11.38 | 0.003* | 0.937 | 0.003* | 0.003* |
|  | Purse-seine | 2 | 11.415 | 0.003* | 0.003* | 0.003* | 0.818 |
|  | Trawling | 2 | 13.345 | 0.001* | 0.003* | 0.003* | 0.041* |
| Adaptive capacity | Muti-gear | 2 | 13.205 | 0.001* | 0.026* | 0.007* | 0.007* |
|  | Purse-seine | 2 | 10.819 | 0.004* | 0.007* | 0.007* | 0.818 |
|  | Trawling | 2 | 10.982 | 0.004* | 0.007* | 0.589 | 0.007* |

| **Supplementary Table S6**. Kruskal-Wallis test and Wilcoxon rank sum test for each component of vulnerability of landings revenue (sensitivity, exposure and adaptive capacity) comparing between areas (North, Centre and South) and fishing fleets (Multi-gear; *Multi*, Purse-seine; *Seine*, Trawling; *Trawl*).* significant p-value (p<0.05). | | | | | | | |
| --- | --- | --- | --- | --- | --- | --- | --- |
| **Landings Revenue** | | **Kruskal-Wallis test** | | | **Wilcoxon test** | | |
| *Fishing fleet comparisons* | | df | H | p-value | Multi-Seine | Multi-Trawl | Seine-Trawl |
| Sensitivity | North | 2 | 13.053 | 0.001* | 0.003* | 0.065 | 0.003* |
|  | Centre | 2 | 15.158 | 0.001* | 0.002* | 0.002* | 0.002* |
|  | South | 2 | 15.158 | 0.001* | 0.002* | 0.002* | 0.002* |
| Exposure | North | 2 | 15.158 | 0.001* | 0.002* | 0.002* | 0.002* |
|  | Centre | 2 | 14.363 | 0.001* | 0.003* | 0.009* | 0.003* |
|  | South | 2 | 15.158 | 0.001* | 0.002* | 0.002* | 0.002* |
| Adaptive capacity | North | 2 | 13.053 | 0.001* | 0.003* | 0.065 | 0.003* |
|  | Centre | 2 | 15.158 | 0.001* | 0.002* | 0.002* | 0.002* |
|  | South | 2 | 12.877 | 0.002* | 0.041* | 0.007* | 0.007* |
| *Area comparisons* | | df | H | p-value | North-Centre | North-South | Centre-South |
| Sensitivity | Muti-gear | 2 | 15.18 | 0.001* | 0.002* | 0.002* | 0.002* |
|  | Purse-seine | 2 | 8.503 | 0.001* | 0.023* | 0.023* | 0.699 |
|  | Trawling | 2 | 13.661 | 0.001* | 0.003* | 0.003* | 0.026* |
| Exposure | Muti-gear | 2 | 12.538 | 0.002* | 0.132 | 0.003* | 0.003* |
|  | Purse-seine | 2 | 11.661 | 0.003* | 0.003* | 0.003* | 0.485 |
|  | Trawling | 2 | 7.029 | 0.030* | 0.310 | 0.013* | 0.310 |
| Adaptive capacity | Muti-gear | 2 | 14.000 | 0.001* | 0.015* | 0.003* | 0.003* |
|  | Purse-seine | 2 | 6.351 | 0.042* | 0.078 | 0.485 | 0.097 |
|  | Trawling | 2 | 15.158 | 0.001* | 0.002* | 0.002* | 0.002* |

| **Supplementary Table S7.** Muli-gear fishery dependence and species vulnerability for each species landed. Only species that had a contribution higher than 3% to the total vulnerability (Vul) of the landings and or to the total landings weight (W) and revenue (Rev) were included in the analysis. Mean values correspond to the average values of landing weight and landing revenue proportion. The categorization in very low (dark green), low (light green), moderate (yellow) and high (light red) are indicated according to the classification proposed in the section 2.3. | | | | | | | | |
| --- | --- | --- | --- | --- | --- | --- | --- | --- |
| **Area** | **Species** | **Fishery Dependence** | | | | **Vulnerability** | | |
|  |  | **W**  **(%)** | **Rev**  **(%)** | **Mean** | **Category** | **Vul.**  **(%)** | **Vul.**  **score** | **Category** |
| North | *Aphanopus carbo* | 5.43 | 6.48 | 5.95 | Low | 3.16 | 0.15 | Very low |
|  | *Cerastoderma edule* | 7.67 | 2.77 | 5.22 | Low | 5.15 | 0.166 | Very low |
|  | *Conger conger* | 3.29 | 3.42 | 3.36 | Very low | 6.90 | 0.539 | Moderate |
|  | *Dicentrarchus labrax* | 1.33 | 5.44 | 3.38 | Very low | 2.15 | 0.418 | Moderate |
|  | *Merluccius merluccius* | 7.69 | 8.04 | 7.87 | Low | 7.60 | 0.256 | Low |
|  | *Octopus vulgaris* | 22.10 | 31.62 | 26.86 | Moderate | 33.09 | 0.39 | Low |
|  | *Raja clavata* | 1.76 | 1.65 | 1.70 | Very low | 3.53 | 0.513 | Moderate |
|  | *Sardina pilchardus* | 12.65 | 4.93 | 8.79 | Low | 12.05 | 0.246 | Low |
|  | *Scomber colias* | 4.08 | 0.74 | 2.41 | Very low | 5.31 | 0.339 | Low |
|  | *Sepia officinalis* | 2.12 | 3.16 | 2.64 | Very low | 1.47 | 0.177 | Very low |
|  | *Solea solea* | 1.54 | 5.14 | 3.34 | Very low | 1.43 | 0.238 | Low |
|  | *Spisula solida* | 3.72 | 3.83 | 3.78 | Very low | -0.98 | -0.067 | Very low |
|  | *Trachurus trachurus* | 10.61 | 5.24 | 7.93 | Low | 10.32 | 0.25 | Low |
|  | *Trisopterus luscus* | 7.19 | 5.16 | 6.17 | Low | 1.40 | 0.05 | Very low |
| Centre | *Aphanopus carbo* | 22.78 | 23.74 | 23.26 | Moderate | 12.73 | 0.169 | Very low |
|  | *Conger conger* | 2.61 | 2.50 | 2.55 | Very low | 4.89 | 0.569 | Moderate |
|  | *Dicentrarchus labrax* | 1.25 | 5.08 | 3.17 | Very low | 1.94 | 0.471 | Moderate |
|  | *Octopus vulgaris* | 15.29 | 25.41 | 20.35 | Moderate | 20.05 | 0.399 | Low |
|  | *Raja clavata* | 1.76 | 1.72 | 1.74 | Very low | 3.15 | 0.547 | Moderate |
|  | *Sardina pilchardus* | 4.64 | 2.61 | 3.62 | Very low | 4.22 | 0.272 | Low |
|  | *Scomber colias* | 25.28 | 2.34 | 13.81 | Low | 30.61 | 0.37 | Low |
|  | *Sepia officinalis* | 5.00 | 8.23 | 6.61 | Low | 3.58 | 0.217 | Low |
|  | *Solea solea* | 1.66 | 7.01 | 4.34 | Very low | 1.21 | 0.221 | Low |
|  | *Sparus aurata* | 0.85 | 3.32 | 2.08 | Very low | 0.92 | 0.33 | Low |
|  | *Trachurus trachurus* | 4.84 | 2.97 | 3.91 | Very low | 4.16 | 0.262 | Low |
| South | *Conger conger* | 2.26 | 1.89 | 2.08 | Very low | 3.76 | 0.562 | Moderate |
|  | *Lophius* spp. | 1.85 | 3.07 | 2.46 | Very low | 1.76 | 0.325 | Low |
|  | *Mullus surmuletus* | 0.96 | 3.59 | 2.28 | Very low | 0.65 | 0.23 | Low |
|  | *Octopus vulgaris* | 35.53 | 49.14 | 42.33 | High | 41.01 | 0.394 | Low |
|  | *Sardina pilchardus* | 10.77 | 5.87 | 8.32 | Low | 8.94 | 0.276 | Low |
|  | *Scomber colias* | 21.48 | 2.43 | 11.95 | Low | 23.53 | 0.367 | Low |
|  | *Sepia officinalis* | 4.20 | 5.98 | 5.09 | Low | 2.75 | 0.223 | Low |
|  | *Trachurus trachurus* | 4.73 | 3.22 | 3.97 | Very low | 3.53 | 0.253 | Low |

| **Supplementary Table S8.** Purse-seine fishery dependence and species vulnerability for each species landed. Only species that had a contribution higher than 3% to the total vulnerability (Vul) of the landings and or to the total landings weight (W) and revenue (Rev) were included in the analysis. Mean values correspond to the average values of landing weight and landing revenue proportion. The categorization in very low (dark green), low (light green), moderate (yellow), high (light red) and very high (red) are indicated according to the classification proposed in the section 2.3. | | | | | | | | |
| --- | --- | --- | --- | --- | --- | --- | --- | --- |
| **Area** | **Species** | **Fisheries Dependence** | | | | **Vulnerability** | | |
|  |  | **W**  **(%)** | **Rev**  **(%)** | **Mean** | **Category** | **Vul.**  **(%)** | **Vul.**  **score** | **Category** |
| North | *Sardina pilchardus* | 55.89 | 72.55 | 64.22 | Very high | 51.41 | 0.246 | Low |
|  | *Scomber colias* | 30.81 | 11.29 | 21.05 | Moderate | 38.14 | 0.339 | Low |
|  | *Trachurus trachurus* | 7.92 | 7.35 | 7.63 | Low | 7.23 | 0.25 | Low |
| Centre | *Sardina pilchardus* | 32.75 | 62.51 | 47.63 | High | 27.91 | 0.272 | Low |
|  | *Scomber colias* | 55.66 | 20.61 | 38.14 | High | 63.13 | 0.37 | Low |
|  | *Trachurus trachurus* | 7.82 | 10.07 | 8.94 | Low | 6.25 | 0.262 | Low |
| South | *Sardina pilchardus* | 32.17 | 61.41 | 46.79 | High | 27.80 | 0.276 | Low |
|  | *Scomber colias* | 56.84 | 17.96 | 37.40 | High | 64.02 | 0.367 | Low |
|  | *Trachurus trachurus* | 6.48 | 9.87 | 8.17 | Low | 5.02 | 0.253 | Low |

| **Supplementary Table S9.** Trawling fishery dependence and species vulnerability for each species landed. Only species that had a contribution higher than 3% to the total vulnerability (Vul) of the landings and or to the total landings weight (W) and revenue (Rev) were included in the analysis. Mean values correspond to the average values of landing weight and landing revenue proportion. The categorization in very low (dark green), low (light green), moderate (yellow) and high (light red) are indicated according to the classification proposed in the section 2.3. | | | | | | | | |
| --- | --- | --- | --- | --- | --- | --- | --- | --- |
| **Area** | **Species** | **Fisheries Dependence** | | | | **Vulnerability** | | |
|  |  | **Land**  **(%)** | **Rev**  **(%)** | **Mean** | **Category** | **Vul.**  **(%)** | **Vul.**  **score** | **Category** |
| North | *Loligo vulgaris* | 1.20 | 5.42 | 3.31 | Very low | 1.81 | 0.386 | Low |
|  | *Merluccius merluccius* | 5.10 | 8.48 | 6.79 | Low | 5.12 | 0.256 | Low |
|  | *Micromesistius poutassou* | 5.48 | 2.45 | 3.97 | Very low | 4.33 | 0.202 | Low |
|  | *Octopus vulgaris* | 2.39 | 5.91 | 4.15 | Very low | 3.64 | 0.39 | Low |
|  | *Pagellus acarne* | 2.98 | 6.35 | 4.67 | Very low | 2.76 | 0.236 | Low |
|  | *Parapenaeus longirostris* | 0.86 | 6.34 | 3.60 | Very low | 0.29 | 0.086 | Very low |
|  | *Scomber colias* | 5.17 | 1.25 | 3.21 | Very low | 6.82 | 0.339 | Low |
|  | *Scomber scombrus* | 3.20 | 1.92 | 2.56 | Very low | 2.58 | 0.206 | Low |
|  | *Trachurus picturatus* | 10.26 | 2.95 | 6.61 | Low | 12.11 | 0.303 | Low |
|  | *Trachurus trachurus* | 54.77 | 44.79 | 49.78 | High | 53.77 | 0.25 | Low |
|  | *Trisopterus luscus* | 3.97 | 3.51 | 3.74 | Very low | 0.78 | 0.05 | Very low |
| Centre | *Lophius* spp. | 2.51 | 4.67 | 3.59 | Very low | 3.57 | 0.315 | Low |
|  | *Merluccius merluccius* | 2.41 | 2.25 | 2.33 | Very low | 3.04 | 0.275 | Low |
|  | *Micromesistius poutassou* | 71.42 | 9.93 | 40.68 | High | 66.30 | 0.202 | Low |
|  | *Nephrops norvegicus* | 6.05 | 30.19 | 18.12 | Moderate | 10.20 | 0.372 | Low |
|  | *Parapenaeus longirostris* | 10.37 | 48.94 | 29.65 | Moderate | 8.09 | 0.173 | Very low |
|  | *Trachurus trachurus* | 3.07 | 0.71 | 1.89 | Very low | 3.57 | 0.262 | Low |
| South | *Lophius* spp. | 4.46 | 4.19 | 4.33 | Very low | 6.16 | 0.235 | Low |
|  | *Merluccius merluccius* | 6.31 | 2.49 | 4.40 | Very low | 6.88 | 0.256 | Low |
|  | *Micromesistius poutassou* | 36.38 | 2.66 | 19.52 | Moderate | 30.75 | 0.202 | Low |
|  | *Nephrops norvegicus* | 9.90 | 23.99 | 16.95 | Moderate | 15.62 | 0.243 | Low |
|  | *Octopus vulgaris* | 4.41 | 3.05 | 3.73 | Very low | 7.40 | 0.39 | Low |
|  | *Parapenaeus longirostris* | 25.24 | 56.94 | 41.09 | High | 18.32 | 0.086 | Very low |
|  | *Sepia officinalis* | 3.21 | 2.23 | 2.72 | Very low | 3.08 | 0.177 | Very low |
|  | *Trachurus trachurus* | 3.31 | 0.49 | 1.90 | Very low | 3.56 | 0.25 | Low |


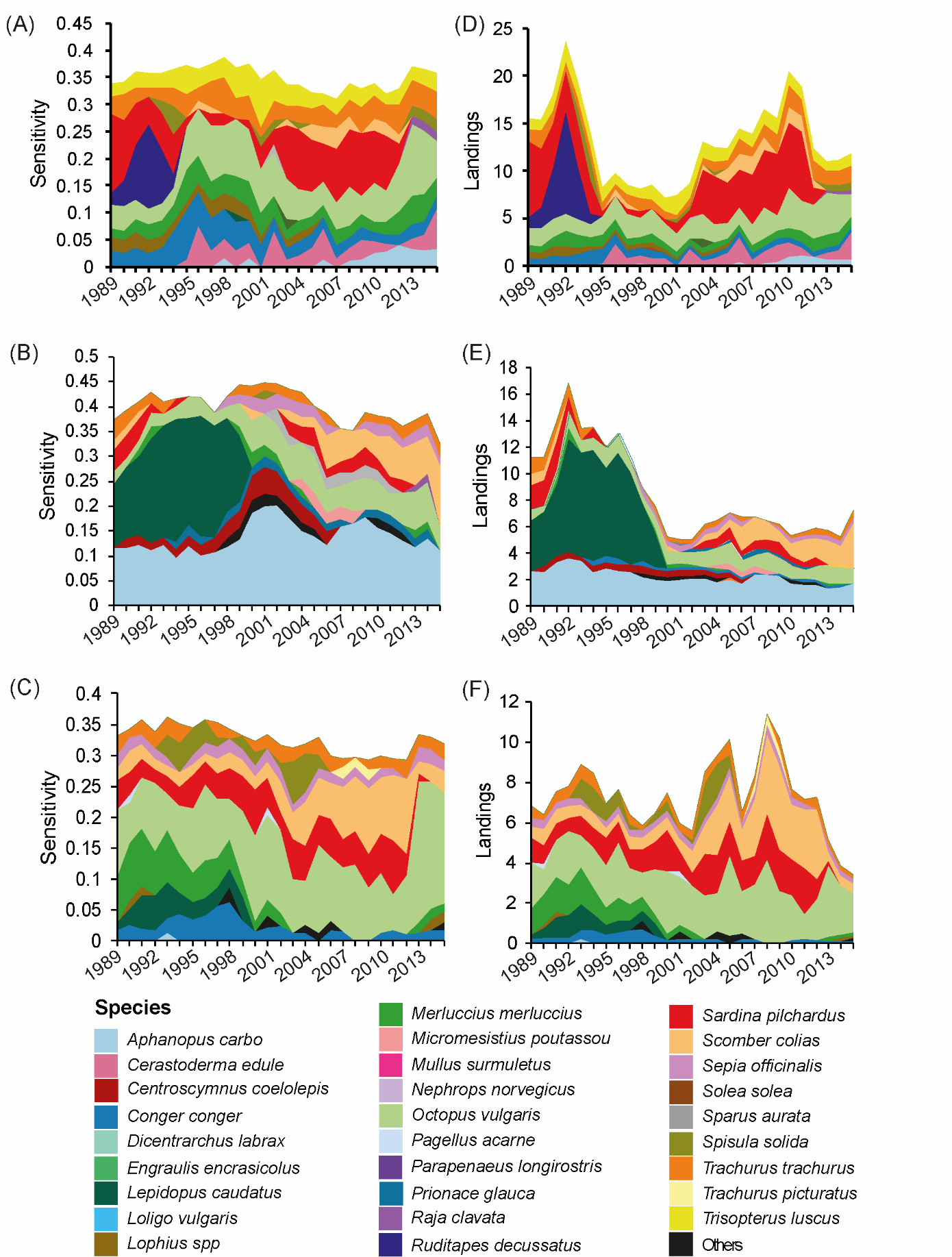
 **Supplementary Figure S1**. Multi-gear fishery sensitivity contribution (A, B, C) and landings (thousand tons; D, E, F) by year for the main species that contributed to the total sensitivity (>3%) in the north (A,D), centre (B,E) and south (C, F) area.


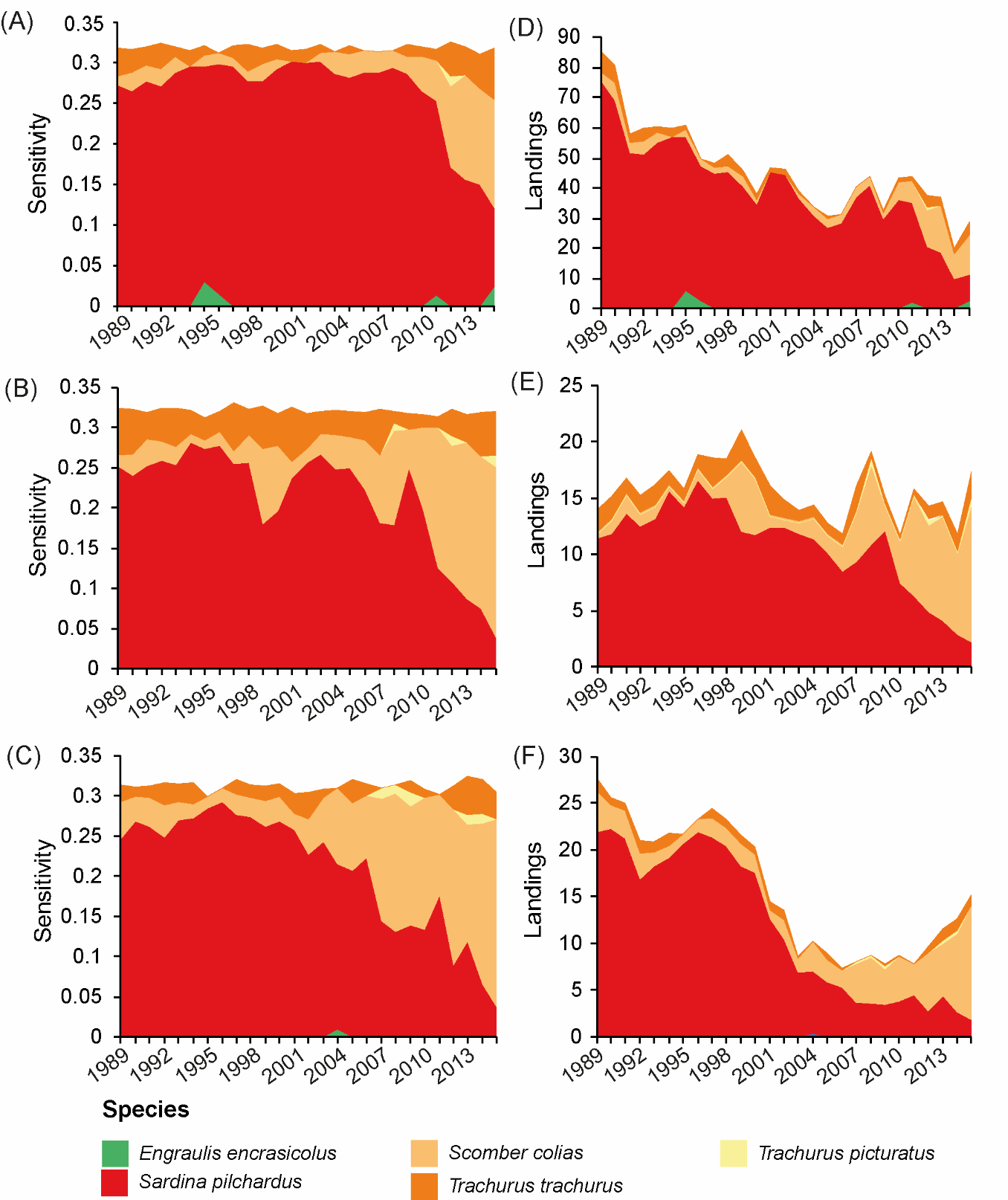


**Supplementary Figure S2**. Purse-seine fishery sensitivity contribution (A, B, C) and landings (thousand tons; D, E, F) by year for the main species that contributed to the total sensitivity (>3%) in the north (A,D), centre (B,E) and south (C, F) area.


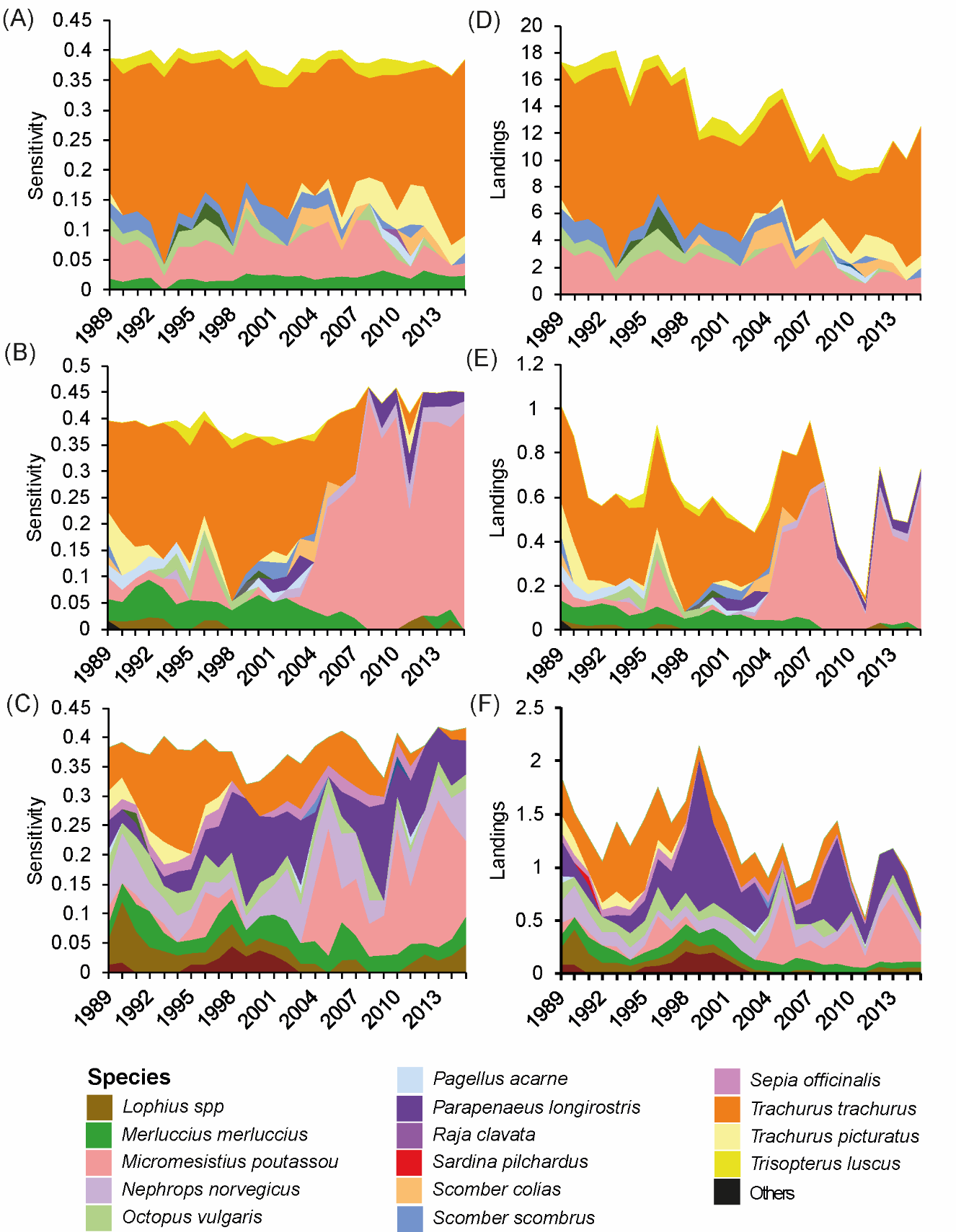


**Supplementary Figure S3**. Trawling fishery sensitivity contribution (A, B, C) and landings (thousand tons; D, E, F) by year for the main species that contributed to the total sensitivity (>3%) in the north (A,D), centre (B,E) and south (C, F) area.


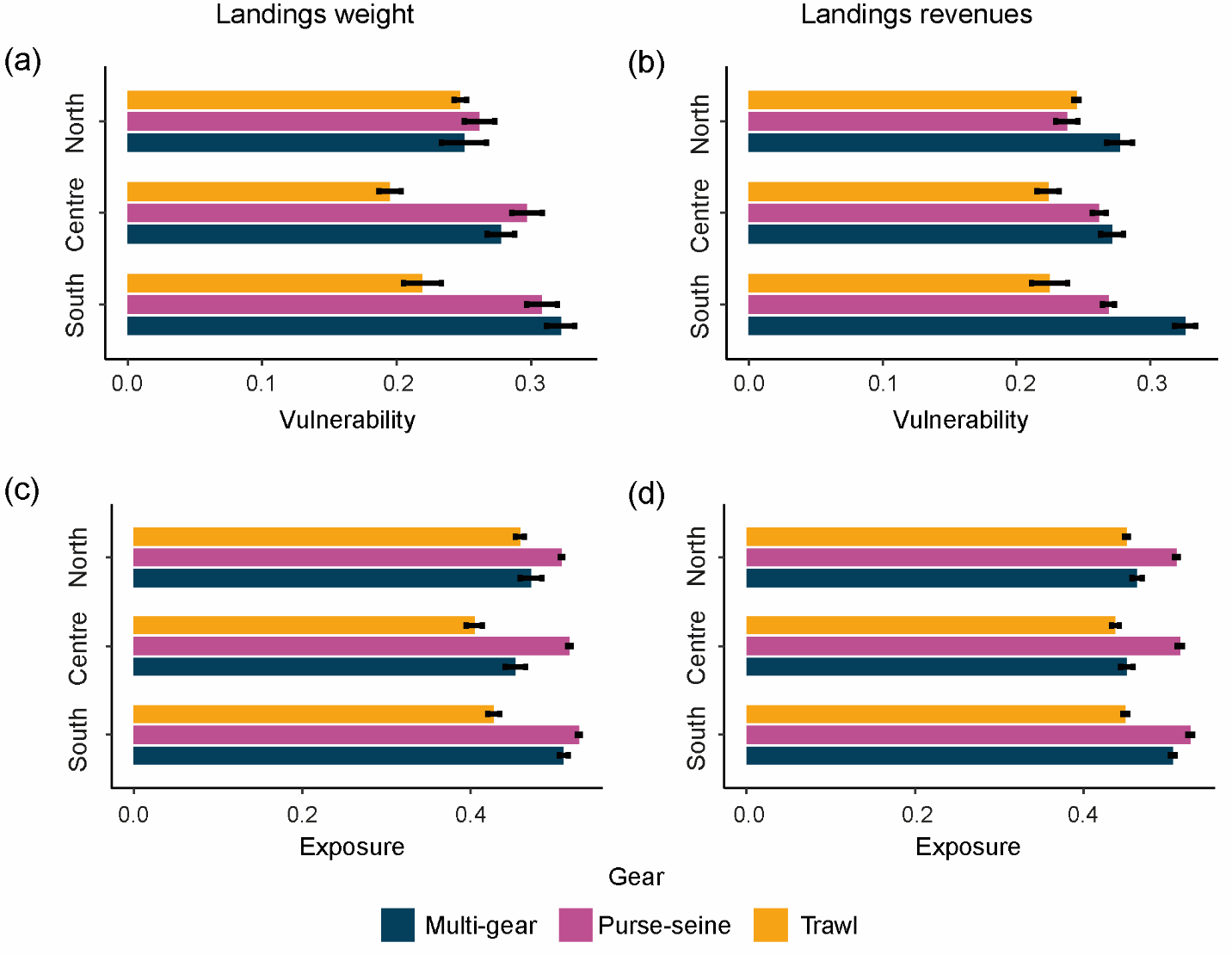


**Supplementary Figure S4**. Ecological vulnerability of the landings weight (a) and economic revenue (b) and ecological exposure of the landings weight (c) and ecoomic revenue (d) by gear type and area calculated for the average period 2010-2015 and RCP 4.5. Each gear type is represented with a different colour.
